# From genomic spectrum of NTRK genes to adverse effects of its inhibitors, a comprehensive genome-based and real-world pharmacovigilance analysis

**DOI:** 10.3389/fphar.2024.1329409

**Published:** 2024-01-31

**Authors:** Zhiwei Cui, Zhen Zhai, De Xie, Lihui Wang, Feiyan Cheng, Siyu Lou, Fan Zou, Rumeng Pan, Shixue Chang, Haoyan Yao, Jing She, Yidan Zhang, Xinyuan Yang

**Affiliations:** ^1^ Department of Obstetrics and Gynecology, The First Affiliated Hospital of Xi’an Jiaotong University, Xi’an, China; ^2^ Department of Oncology, The Second Affiliated Hospital of Xi’an Jiaotong University, Xi’an, China; ^3^ Department of Endocrinology, Xiang’an Hospital of Xiamen University, School of Medicine, Xiamen University, Xiamen, Fujian, China; ^4^ Department of Respiratory and Critical Care Medicine, Affiliated Hospital of Zunyi Medical University, Zunyi, China; ^5^ Center for Translational Medicine, The First Affiliated Hospital of Xi’an Jiaotong University, Xi’an, China

**Keywords:** NTRK, gene fusion, entrectinib, larotrectinib, FAERS, adverse drug event, pharmacovigilance

## Abstract

**Introduction:** The discovery of neurotrophic tyrosine receptor kinase (*NTRK*) gene fusions has facilitated the development of precision oncology. Two first-generation *NTRK* inhibitors (larotrectinib and entrectinib) are currently approved for the treatment of patients with solid tumors harboring *NTRK* gene fusions. Nevertheless, comprehensive *NTRK* profiling at the pan-cancer genomic level and real-world studies pertaining to the adverse events of *NTRK* inhibitors are lacking.

**Methods:** We characterize the genome of *NTRK* at the pan-cancer level through multi-omics databases such as The Cancer Genome Atlas (TCGA). Through the FDA Adverse Event Reporting System (FAERS) database, we collect reports of entrectinib and larotrectinib-induced adverse events and perform a pharmacovigilance analysis using various disproportionality methods.

**Results:** *NTRK1/2/3* expression is lower in most tumor tissues, while they have higher methylation levels. *NTRK* gene expression has prognostic value in some cancer types, such as breast invasive carcinoma (BRCA). The cancer type with highest *NTRK* alteration frequency is skin cutaneous melanoma (SKCM) (31.98%). Thyroid carcinoma (THCA) has the largest number of *NTRK* fusion cases, and the most common fusion pair is *ETV6-NTRK3*. Adverse drug events (ADEs) obtained from the FAERS database for larotrectinib and entrectinib are 524 and 563, respectively. At the System Organ Class (SOC) level, both drugs have positive signal value for “nervous system disorder”. Other positive signals for entrectinib include “cardiac disorders”, “metabolism and nutrition disorders”, while for larotrectinib, it is “hepatobiliary disorders”. The unexpected signals are also listed in detail. ADEs of the two *NTRK* inhibitors mainly occur in the first month. The median onset time of ADEs for entrectinib and larotrectinib was 16 days (interquartile range [IQR] 6–86.5) and 44 days ([IQR] 7–136), respectively.

**Conclusion:** Our analysis provides a broad molecular view of the *NTRK* family. The real-world adverse drug event analysis of entrectinib and larotrectinib contributes to more refined medication management.

## 1 Introduction

Members of the tropomyosin receptor kinase (TRK) family include the TRKA, TRKB, and TRKC proteins, encoded by the neurotrophic tyrosine kinase receptor 1 (*NTRK1*), *NTRK2*, and *NTRK3* genes, respectively (Khotskaya et al., 2017). Activation of neurotrophic factors, which are specific ligands for TRK receptors, triggers the activation of various downstream signaling cascades. These cascades include the mitogen-activated protein kinase (MAPK), phosphatidylinositol-3-kinase (PI3K), and phospholipase C-γ (PLC-γ) pathways. These pathways have significant effects on essential biological processes such as neuronal cell differentiation, survival, and proliferation (Bhangoo and Sigal, 2019; Jiang et al., 2022). Alterations in *NTRK* genes are closely associated with both tumor initiation and progression. Among these alterations, *NTRK* gene fusions are the most well-characterized aberrations. These gene fusions possess oncogenic properties as they promote tumorigenesis by constitutively activating downstream cell growth and proliferation pathways (Khotskaya et al., 2017; Cocco et al., 2018; Okamura et al., 2018). In addition to gene fusions, several studies have also characterized the genomic profile of *NTRK*. Light et al. reported that *NTRK1/3* were highly expressed in neuroblastoma patients with a better prognosis, whereas *NTRK2* was highly expressed in neuroblastoma patients with a poor prognosis (Light et al., 2012). Additionally, in 83 clinical samples of triple-negative breast cancers, *NTRK1/2/3* were found to exhibit varying degrees of copy number gain and amplification (Zito Marino et al., 2023). However, these aforementioned studies are limited by focusing on specific cancer types and small sample sizes, highlighting the critical need for comprehensive analyses across multiple tumor types to investigate their functions.

The US Food and Drug Administration (FDA) has approved the first generation of *NTRK* inhibitors, which have shown favorable clinical outcomes. One such inhibitor is larotrectinib, a potent and highly selective small molecule inhibitor targeting three TRK proteins. Larotrectinib is prescribed for the treatment of patients with solid tumors who have *NTRK* gene fusions and have experienced disease progression after alternative therapies (Scott, 2019). Its recommended phase 2 dose is 100 mg twice daily (Laetsch et al., 2018). Whereas entrectinib is mainly used for the treatment of locally advanced or metastatic solid tumors harboring fusion mutations in the *NTRK1/2/3*, C-ros oncogene 1 (*ROS1*) and anaplastic lymphoma kinase (*ALK*) genes. The recommended adult dose of entrectinib is 600 mg/d, while the pediatric dose is based on body surface area (Frampton, 2021). The main difference between the two drugs is that entrectinib is indicated for adult and pediatric patients aged ≥12 years, while larotrectinib is indicated for adult and pediatric patients of all ages (Frampton, 2021). Both drugs prolong metastasis-free survival and overall survival and maintain health-related quality of life in patients with solid tumors (Drilon et al., 2022; Jiang et al., 2022). Adverse events were predominantly reported during clinical trials for both drugs, primarily in grades 1–2, such as fatigue and dizziness. Additionally, a variable proportion of patients (ranging from 2% to 40%) also experienced grade 3–4 adverse events, including anaemia and elevated aminotransferases (Doebele et al., 2020; Hong et al., 2020). However, the majority of clinical trials were unable to identify new signals of adverse drug reactions due to the limited number of patients included and the short duration of follow-up (Doz et al., 2022). Liguori et al. recently collected and descriptively analyzed reports of adverse events following the introduction of two first-generation *NTRK* inhibitors (Liguori et al., 2023). Nevertheless, there is still a lack of disproportionality analysis of the adverse reaction signals of the two first-generation *NTRK* inhibitors, identification of new adverse reaction signals, and detailed comparisons of the safety of the two drugs.

**FIGURE 1:**
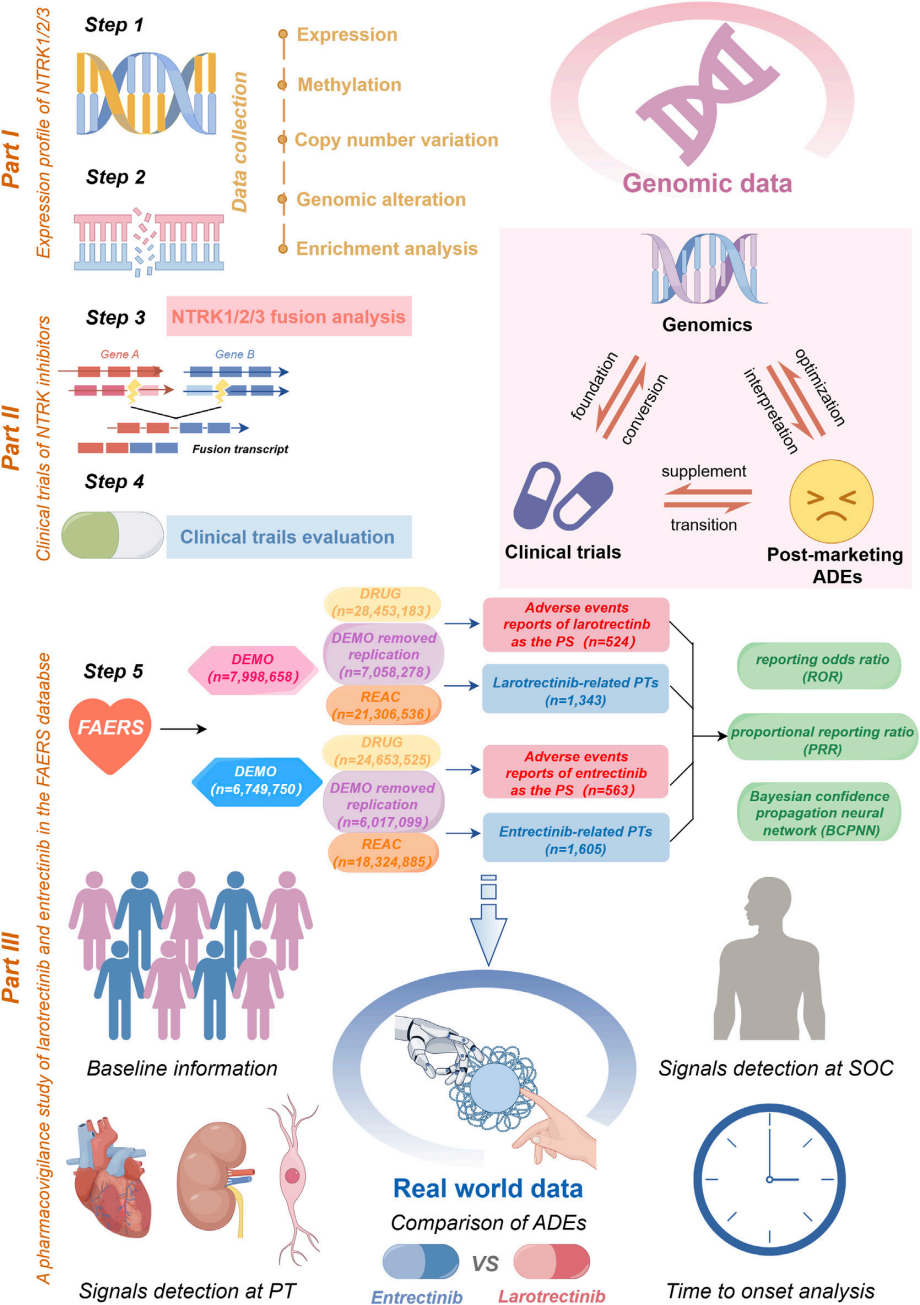
A flow chart of the whole study. In part I, we acquired *NTRK* expression profiling data and conducted an analysis of its mRNA expression, methylation, and CNV (step 1). Subsequently, we delved into the genomic alterations of *NTRK*, co-mutation pathways (step 2), and emphasized *NTRK* fusions (step 3). The part II involved a comprehensive review of the clinical trials of *NTRK* inhibitors (step 4). In part III, a safety assessment of the first-generation *NTRK* inhibitors was carried out utilizing real-world ADE reports sourced from the FAERS database (step 5). This encompassed data cleaning, baseline information description, signal detection, and time to onset analysis. Furthermore, we provided a summarization of the interrelationships between the three different data sources (top right of figure). CNV, copy number variation; FAERS, FDA Adverse Event Reporting System; SOC, System Organ Class; PT, preferred term; ADE, adverse drug event; PS, primary suspect.

Evidence at the genomic level plays a crucial role in the foundation of drug design. Drugs with confirmed targets from human genetic studies are more likely to be successfully marketed than those lacking such evidence (Roden et al., 2019). Furthermore, mutations and epigenetic modifications of drug target genes can significantly and consistently alter cellular gene expression patterns and are strongly associated with drug efficacy and adverse drug reactions (Brockmöller and Tzvetkov, 2008; Borges et al., 2021). For instance, fibroblast growth factor receptor 2 (*FGFR2*) rearrangements in cholangiocarcinoma predict tumor sensitivity to *FGFR2* inhibitors (Vogel et al., 2023). Clinical trials serve as a vital pre-market assessment of drugs and represent an important step in translating genomic evidence into clinical practice (Joffe et al., 2017). However, due to the relatively low exposure of clinical trials with clear inclusion criteria and drug treatment conditions, rare but serious adverse drug reactions are usually detected after marketing authorization and increased population exposure (Ehmann et al., 2014). Therefore, post-market real-world vigilance data on drugs are of paramount importance, complementing evidence from pharmacoepidemiology (Sabaté and Montané, 2023). By integrating data from three sources-genomics, clinical trials, and real-world pharmacovigilance, a comprehensive view is provided, which is not available from any independent data source (Figure 1). This approach also effectively overcomes the limitations of analyzing each data type separately (Jing et al., 2021).

This study aimed to characterize the molecular signature of *NTRK* using a large genomic dataset covering diverse tumor types, and to conduct a comprehensive analysis of the safety of first-generation *NTRK* inhibitors based on real-world adverse drug event (ADE) data. Firstly, we conducted a systematic analysis of *NTRK* expression, methylation, and genomic alterations across various cancer types. Additionally, we investigated *NTRK* fusions and examined the current status of clinical trials for the first-generation *NTRK* inhibitors, including larotrectinib and entrectinib. Lastly, we compared the differences in ADEs between the two drugs and assessed the time of onset for these ADEs. The integration of data from various sources in our study offers a comprehensive perspective on the *NTRK* gene.

## 2 Materials and methods

### 2.1 Genomics data collection and processing

The RNA sequencing data from a pan-cancer microarray (n = 15,776) was obtained from the UCSC Xena browser (https://xenabrowser.net/datapages/) on 1 August 2023. This dataset integrates various cancerous and normal tissues obtained from The Cancer Genome Atlas (TCGA) and the Genotype-Tissue Expression (GTEx) (Goldman et al., 2020). Secondly, these data were filtered and normalized before subsequent analyses were performed (Vivian et al., 2017; Zhao et al., 2020). For detailed procedures, please refer to a previously published article of us (Cui et al., 2023).

To assess the correlation between *NTRK1/2/3* gene expression (with the median value as the cut-off) and patient prognosis, we utilized univariate Cox proportional hazards regression analysis to calculate hazard ratios (HR) and corresponding *p*-values. Patient survival indicators examined in this study comprised overall survival (OS), disease-specific survival (DSS), and progression-free interval (PFI). OS was defined as death from any cause, with surviving patients censored at the last follow-up. DSS was determined based on deaths attributed to a specific disease, and PFI represented the duration between the date of initial treatment in the randomized group and disease recurrence (Ekmekcioglu et al., 2016; Liu et al., 2018). During this process, key R packages was utilized, including “survival (version 3.3.1)” and “ggplot (version 3.3.6)”.

### 2.2 Genomic alteration and enrichment analysis

Gene Set Cancer Analysis (GSCA) (http://bioinfo.life.hust.edu.cn/GSCA/#/) is an integrative platform for genomic cancer analysis (Liu et al., 2023). We employed the “Mutation” module to investigate the association between *NTRK1/2/3* mRNA expression and copy number variation (CNV) as well as methylation across various tumor types. Additionally, we utilized the “Expression” module to explore the correlation between gene expression and pathway activity. All analyses were conducted on 1 August 2023.

The cBioPortal (https://www.cbioportal.org/) provides an open access to the interactive exploration of multidimensional cancer genomics data (Cerami et al., 2012; Gao et al., 2013). To systematically analyze the genomic alterations of *NTRK1/2/3* genes across 32 different cancer types, we selected the “TCGA Pan Cancer Atlas Studies” cohort, which consisted of a total of 10,953 patients (including 10,967 samples) for further investigation. This cohort was chosen from the “Cancer Types Summary” and “Mutations” modules. To identify the most frequently altered genes, we utilized the “Genomic alterations” option under the “Comparison/Survival” module. Specifically, we focused on genes with alteration cases surpassing 100 in the altered group (totaling 2,799 genes) that were found to have q-values<0.01. The top 20 genes with the highest alteration frequencies in the altered group were subsequently visualized using a bar graph. To gain insights into the potential functions of these genes, a functional enrichment analysis was conducted. This analysis was performed using the R packages “clusterProfiler” (version 4.4.4) for enrichment analysis and “org.Hs.e.g.,.db” (version 3.1.0) for ID conversion (Yu et al., 2012). The *p*-values were adjusted using BH correction. All operational processes were conducted on 4 August 2023.

### 2.3 Fusion gene analysis

The fusion gene data of *NTRK1/2/3* were retrieved from the TCGA Fusion Gene Database (https://www.tumorfusions.org/) on 5 August 2023. The database’s pipeline for RNA sequencing Data Analysis (PRADA) allowed us to identify fusion transcripts comprehensively and with high confidence, and provided a list of authentic fusion genes across 33 TCGA cancer types (Hu et al., 2018). The database defines four tiers to rank the screened fusion transcripts according to the abundance of supporting evidence. From tier 1 to tier 4 indicates strong to weak confidence levels.

### 2.4 My cancer genome

The My Cancer Genome (MCG) database were launched in 2011 to guide clinicians in applying genomic test results to the treatment of cancer patients (Jain et al., 2020; Jain et al., 2021). Under the “Drugs” module, we selected two first-generation *NTRK* inhibitors (including larotrectinib and entrectinib) and systematically surveyed the top biomarker inclusion criteria for open clinical trials investigating these two drugs (5 August 2023).

### 2.5 Adverse drug event data collection and deduplication

Adverse drug events (ADEs) are unintended and harmful effects of medication use, and they commonly result in unplanned hospitalizations and fatalities (Bailey et al., 2016; Visacri et al., 2022). To assess the post-marketing safety of entrectinib and larotrectinib, we conducted a retrospective pharmacovigilance study with ADEs data extracted from the FAERS database. The FAERS data consists of seven datasets: demographic and administrative information (DEMO), drug information (DRUG), adverse drug reaction information (REAC), patient outcomes information (OUCT), reported sources (RPSR), drug therapy start dates and end dates (THER), and indications for drug administration (INDI). Considering the different timing of FDA approval for marketing of the two drugs, we collected information on all ADEs from the FAERS database from 2018 Q4 to 2023 Q1 (for larotrectinib, approved for marketing in 2018 Q4), and from 2019 Q3 to 2023 Q1 (for entrectinib, approved for marketing in 2019 Q3), respectively. As the database is updated on a quarterly basis, it is inevitable that there will be duplicates of previous published reports. In accordance with FDA recommendations, we performed deduplication prior to statistical analyses based on the following criteria: if CASEIDs were the same, the most recent FDA_DT was selected; if CASEIDs and FDA_DTs were the same, the higher PRIMARYIDs were selected (Shu et al., 2022). In this study, “larotrectinib” (brand name: VITRAKVI), and “entrectinib” (brand name: ROZLYTREK) were used to recognize records related to the two *NTRK* inhibitors. To analyze the role of drugs in ADEs, the drugs involved in ADE reports were classified as: primary suspect (PS), which denotes the primary drug considered to have possibly caused the ADE; second suspect (SS), which denotes the secondary drug considered to have possibly caused the ADE; concomitant (C), indicating the concurrent use of other drugs associated with the ADE; and interacting (I), representing possible drug interactions associated with the ADE. To improve accuracy, role codes for ADEs are reserved for entrectinib and larotrectinib as the PS drugs only, which resulted in ADEs associated with entrectinib and larotrectinib dosing being 563 and 524, respectively (Wu et al., 2023). System Organ Class (SOC) was the highest level of terminology in the Medical Dictionary for Regulatory Activities (MedDRA, version 26.0). All ADEs in the report were coded according to the preferred terms (PTs). Consequently, 1,343 larotrectinib-related PTs and 1,605 entrectinib-related PTs were screened out.

### 2.6 Data mining algorithm and statistical analysis

In pharmacovigilance studies, the term “signal” denotes a statistical association between a drug and an adverse event or drug-related adverse event (Hauben and Aronson, 2009; Sakaeda et al., 2013). Disproportionality analysis is a vital analytical method used to identify signals of adverse reactions associated with drugs, as well as to compare the occurrence of adverse reactions between a specific drug and all other drugs. This analysis helps uncover drug-related adverse reactions by evaluating the proportion of these reactions in relation to the overall adverse reaction pool (Montastruc et al., 2011). Therefore, in this study, we employed disproportionality analysis to identify potential correlations between the use of two *NTRK* inhibitors and all ADEs. We utilized two non-Bayesian algorithms, namely, the reporting odds ratio (ROR) and the proportional reporting ratio (PRR), along with a Bayesian algorithm known as the Bayesian confidence propagation neural network (BCPNN) (Montastruc et al., 2011). The Bayesian method (BCPNN) have sufficient power to detect unique signals even when few ADEs are reported for a drug (Bate, 2007). Generally, a higher parameter value indicates a stronger signal value. The specific formulas and criteria for detecting positive safety signals using the three algorithms are presented in Table 1. To enhance the reliability of our findings, we considered only adverse drug events (ADEs) with positive signal values that satisfied all three algorithms simultaneously. Furthermore, we excluded ADEs related to drug indications to ensure clarity in our presentation. Unexpected signals were determined as positive ADEs that were detected but not listed in the drug instruction. The primary analyses of this study are depicted in Figure 1. All data processing and statistical analyses were performed using SAS 9.4, Microsoft Excel 2019, and R software (version 4.2.1).

**TABLE 1:** Three disproportionality algorithms for assessing potential associations between *NTRK* inhibitors and ADEs.

Algorithms	Equation	Criteria
ROR	ROR = ad/bc, 95%CI = e^ln(ROR)±1.96 (1/a+1/b+1/c+1/d)^0.5^	lower limit of 95% CI > 1, N ≥ 3
PRR	PRR = [a (c + d)]/[c (a+b)]	PRR≥2, χ^2^ ≥ 4, N ≥ 3
	χ2 = [(ad-bc)^2](a+b + c + d)/[(a+b)(c + d)(a+c)(b + d)]	
BCPNN	IC = log_2_a (a+b + c + d)/[(a+c)(a+b)], 95%CI = E (IC) ± 2V(IC)^0.5	IC025 > 0

a, number of reports that contain both targeted drug and targeted drug adverse reactions; b, number of reports of other drug adverse reactions that contain the targeted drug; c, number of reports of targeted drug adverse reactions that contain other drugs; d, number of reports that contain other drugs and other drug adverse reactions. ROR, reporting odds ratio; PRR, proportional reporting ratio; BCPNN, Bayesian confidence propagation neural network; 95% CI, 95% confidence interval; N, reports number; χ2, chi-squared; IC, information component; IC025, the lower limit of the 95% CI of IC; E (IC), the IC expectations; V(IC), the variance of IC

### 2.7 Time to onset analysis

The time to onset (TTO) for ADEs associated with *NTRK* inhibitor dosing was calculated as the interval between the ADEs onset date (EVENT_DT) within the DEMO file, and the start date of *NTRK* inhibitor dosing (START_DT) within the THER file. The exclusion criteria involved removing inaccurate or missing dates and cases where the ADEs onset date (EVENT_DT) preceded the start date of *NTRK* inhibitor dosing (START_DT). By utilizing the Weibull distribution, we could identify and estimate the increase or decrease in the incidence of ADE risk over time. The Weibull distribution employs two parameters, scale (α) and shape (β), to determine its shape (Kazi et al., 2020).

## 3 Results

### 3.1 Expression and clinical analysis of *NTRK*1/2/3 in pan-cancer

**FIGURE 2:**
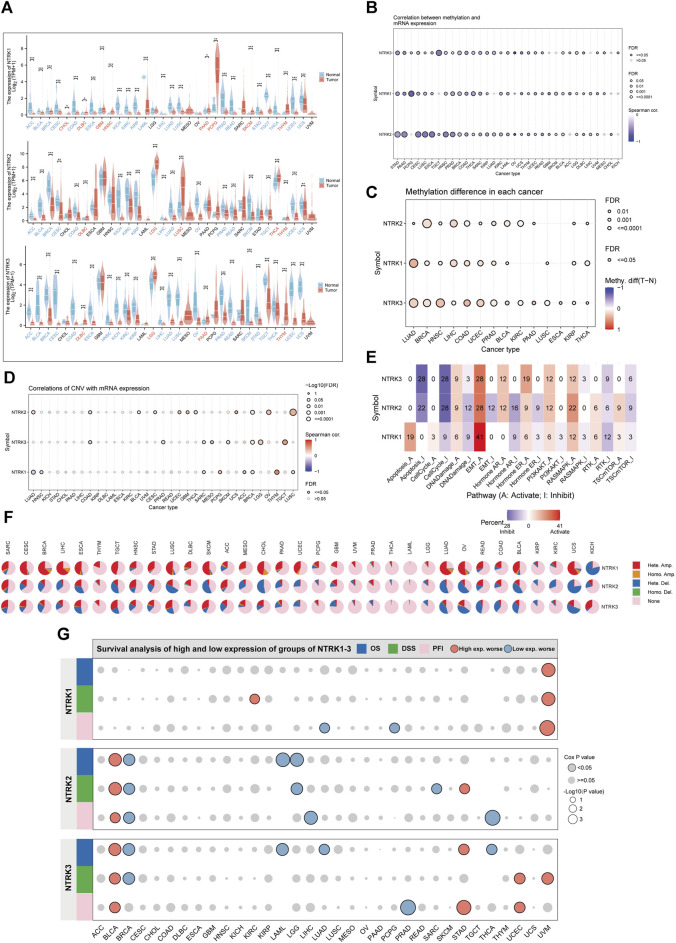
Genomic characterization of *NTRK* genes at the pan-cancer level. **(A)**. Differential mRNA expression of *NTRK1/2/3* in tumor and corresponding normal tissues. Gene expression is compared after Log2 (TPM+1) transformed. **(B)**. Bubble plots demonstrate the correlation between *NTRK* genes expression and methylation levels. The bubble size positively correlates with the significance of FDR. The black outline border indicates FDR≤0.05. **(C)**. Bubble plots depict the methylation differences of *NTRK* genes between tumor and normal samples. Blue dots and red color indicate methylation downregulation and upregulation in tumors, respectively. **(D)**. Figure summarizes the correlations between CNV and *NTRK* genes expression in pan-cancer. **(E)**. Percentage of cancers in which *NTRK1/2/3* expression has a potential impact on pathway activity is shown. Pathway A and I represent activation or inhibition of this pathway. **(F)**. Pie chart showing the percentage of different types of CNV in *NTRK1/2/3* in a given cancer, with different colors representing different CNV types. **(G)**. The relationship between *NTRK* genes expression and patient prognosis (OS, DSS, PFI) in pan-cancer. TPM, transcripts per million; FDR, false discovery rate; CNV, copy number variation; OS, overall survival; DSS, disease-specific survival; PFI, progression-free interval. *p < 0.05. **p < 0.01. ***p < 0.001. ns, no significance.

To gain a comprehensive understanding of *NTRK* genomic mRNA expression, we initially employed data from the TCGA and GTEx databases. *NTRK* mRNA expression was generally downregulated in tumor tissues compared to corresponding normal tissues. For *NTRK1*, *NTRK2* and *NTRK3*, the number of tumors with significantly reduced expression was 20, 17, and 22, respectively (Figure 2A). Notably, there was a consistent trend towards significant downregulation of three *NTRK* genes expression in 15 tumor types (*p* < 0.05). However, in lymphoid neoplasm diffuse large B-cell lymphoma (DLBC) and thymoma (THYM), they were significantly upregulated. The full names of the 33 cancer abbreviations were shown in Supplementary Table S1.

There is accumulating evidence indicating that both CNV and DNA methylation reshapes gene expression repertoire in cancers (Valsesia et al., 2012; Arechederra et al., 2018; Hammam et al., 2020; Ji et al., 2020). We therefore explored the relationship between *NTRK* genes expression and CNV, DNA methylation in different tumor types. Figure 2B indicated that in most tumor types, methylation levels and *NTRK1/2/3* gene expression values were significantly negatively correlated (Spearman’s correlation<0, false discovery rate [FDR]<0.05). Moreover, we compared the methylation difference between tumor and normal and found that methylation of the *NTRK* genes was upregulated in various types, such as lung adenocarcinoma (LUAD), breast invasive carcinoma (BRCA), and head and neck squamous cell carcinoma (HNSC) (Figure 2C). Additionally, we evaluated the relationship between CNV and *NTRK* genes expression and observed a significant correlation in some tumors such as sarcoma (SARC), BRCA, and testicular germ cell tumors (TCGT) (Figure 2D). The comprehensive constitution of the heterozygous/homozygous CNV of *NTRK1/2/3* in each cancer was displayed in Figure 2F.

We also analyzed the roles of *NTRK* genes in cancer pathways. The results suggested that *NTRK1* had a relatively strong activation of apoptosis and a strong activation of epithelial-mesenchymal transition (EMT). *NTRK2* and *NTRK3* shared similar pathway activity. They exhibited strong inhibitory effect on apoptosis and cell cycle, and strong activation of EMT. Overall, *NTRK* genes had strong activating effect on EMT and potent inhibitory effect on the cell cycle (Figure 2E). The relationship between *NTRK* genes expression and patient prognosis was evaluated, as well. Increased *NTRK1* expression was a risk factor for uveal melanoma (UVM), and high *NTRK2* and *NTRK3* expression was associated deceased OS, DSS, and PFI in bladder urothelial carcinoma (BLCA). However, elevated *NTRK2* expression was protective in BRCA (Figure 2G).

### 3.2 Somatic mutation analysis of *NTRK* genes

**FIGURE 3:**
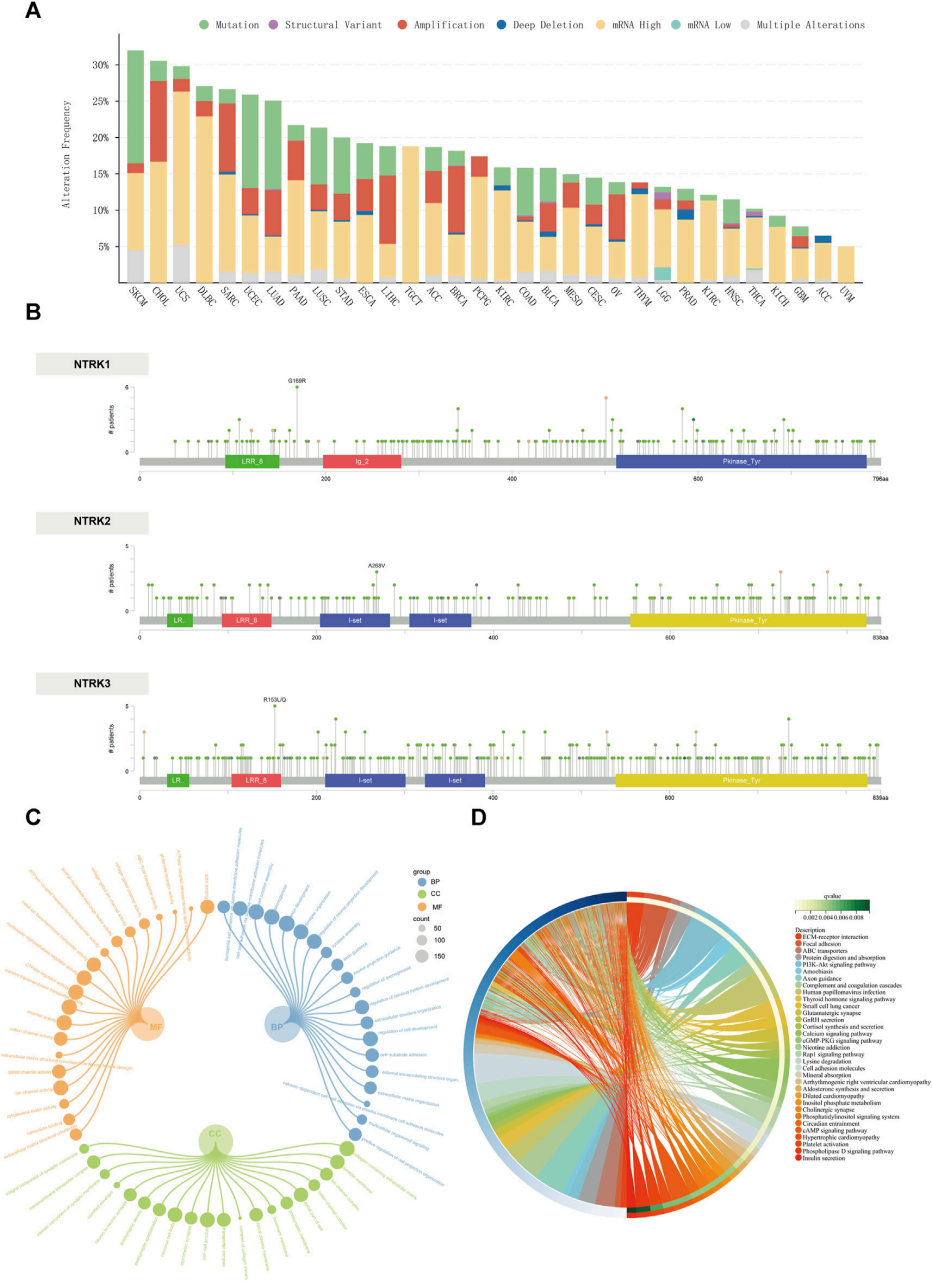
Genomic alterations of the *NTRK* genes at the pan-cancer level. **(A)**. The alteration frequency of *NTRK* genes across 32 TCGA tumor types. **(B)**. Amino acid mutation of in TCGA cancers. The GO and KEGG enrichment analyses of differentially altered genes between *NTRK*-gene-altered and unaltered groups are visualized using circular subgroup plot **(C)** and chord plot **(D)**. GO, gene ontology; BP, biological process; CC, cellular component; MF, molecular function; KEGG, Kyoto Encyclopedia of Genes and Genomes.

As shown in Figure 3A, among the 32 cancer types observed, the frequency of mutations in the *NTRK* genes was not low overall. The frequency of mutations in the *NTRK* gene exceeded 15% in 19 cancer types. The mutation types were mainly composed of mRNA high, mutation, and amplification. The most frequent cancer types with *NTRK* genes alteration were skin cutaneous melanoma (SKCM) (31.98%), cholangiocarcinoma (CHOL) (30.56%), uterine carcinosarcoma (UCS) (29.82%), DLBC (27.08%), and SARC (26.67%). Instead, the mutation frequency of the *NTRK* genes in kidney chromophobe (KICH) (9.23%), glioblastoma multiforme (GBM) (7.77%), adrenocortical carcinoma (ACC) (6.5%), and UVM (5%) was relatively low, not exceeding 10%. Strikingly, all mutations in the *NTRK* gene in TCGT and UVM were mRNA high. In Figure 3B, the type, location and number of *NTRK1/2/3* genetic alterations could further be observed. The hot spot mutation sites G169R (located in the linker) of *NTRK1*, A268V (located in the I-set) of *NTRK2*, and R153L/Q (located in the LRR-8) of *NTRK3* were identified in 6, 3, and 5 patients. In general, the types of mutations at different amino acid sites of the *NTRK1/2/3* gene were primarily missense mutations (Figure 3B). Further analysis of the mutations (Supplementary Figure S1A) revealed that most of the *NTRK1/2/3* mutations were located in the structural domains of the Pkinase-Tyr and LRR-8. Moreover, the I-set domain in *NTRK2/3* also contained close to a quarter of the number of mutations. The Ig-2 domain was specific to *NTRK1* and contained about 5% of the mutations. In the final, we counted the genomic alterations of *NTRK1/2/3* in the TCGA pan-cancer cohort in each patient. Of these, 645, 451, and 584 patients contained alterations in only one of the *NTRK1*, *NTRK2*, and *NTRK3* genes, respectively. There were still 21 individuals with genomic alteration for *NTRK1/2/3* simultaneously (Supplementary Figure S1B).

We also screened for altered genes that were significantly enriched in the *NTRK*-gene-altered group (compared to the unaltered group). The top 20 genes with the highest mutation frequencies in the *NTRK*-gene-altered group were displayed in Supplementary Figure S1C, and the full list of genes was provided in Supplementary Table S2. Genes with significant mutation differences were involved in biological processes including cell junctions, cell adhesion; involved in cellular components including extracellular matrix, synapses and ion channels; involved in molecular functions including cytoskeletal motility and ion channel activity (Figure 3C). Similarly, the signaling pathways and diseases in which these genes may be engaged included hormone synthesis and delivery (thyroid hormones, aldosterone hormone, insulin), some traditional signaling pathways (PI3K/Akt, cGMP-PKG, cAMP, etc.), as well as small-cell lung cancer, and human papillomavirus infection (Figure 3D). All enrichment analysis results were presented in Supplementary Table S3.

### 3.3 Fusion gene analysis

We detected fusion transcripts of the *NTRK1/2/3* genes in pan-cancer through the TCGA Fusion Gene Database with high credibility (Supplementary Figure S2). In total, transcripts of *NTRK* fusion genes were detected in 11 tumor types, especially in thyroid carcinoma (THCA, n = 15), and brain lower grade glioma (LGG, n = 5). The *NTRK3* gene had the highest number of fusion transcripts (n = 18). *NTRK3-ETV6* (n = 10) had the highest percentage of all fusion pairs, especially in THCA (n = 6). Chromosomes 12 and 15 were the major chromosomes for 5′gene junction and 3′gene junction, respectively. The great majority of these *NTRK* fusion transcripts were classified as in-frame transcripts, while two fusion transcripts (*NTRK2-RASEF*, and *FAT1-NTRK3*) were classified as out-of-frame and one (*PAN3-NTRK2*) was classified as 5UTR-CDS. The complete data was available in Supplementary Table S4.

### 3.4 Landscape analysis of clinical trials of larotrectinib and entrectinib

**FIGURE 4:**
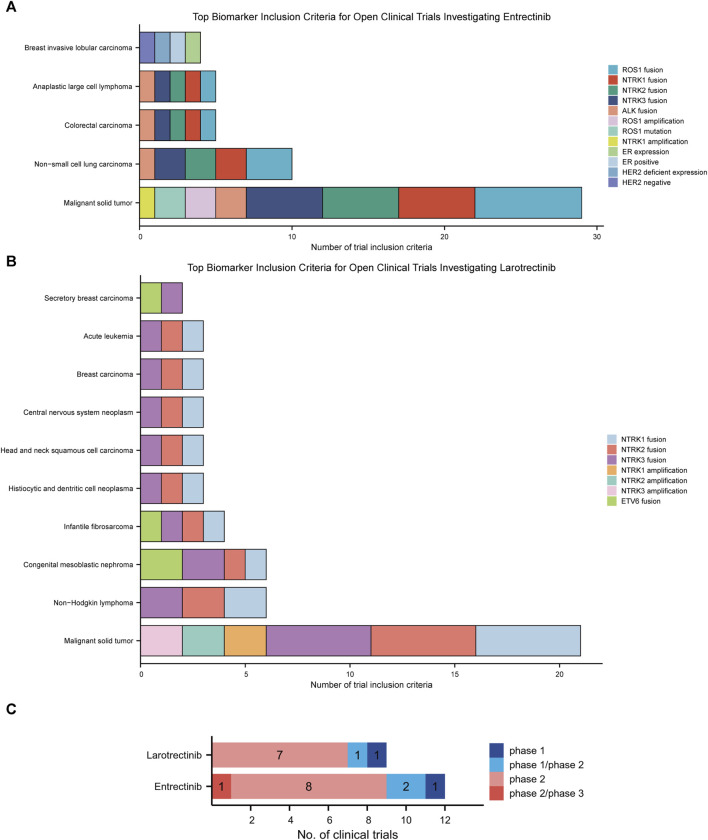
This chart shows the top biomarkers most frequently included in clinical trials investigating larotrectinib **(A)** and entrectinib **(B)**, and the types of cancers associated with those biomarkers. **(C)**. Current clinical trial status of larotrectinib and entrectinib.

First-generation *NTRK* inhibitors (larotrectinib and entrectinib) treat patients with *NTRK* fusion-positive cancers with high remission rates, irrespective of tumor histology (Cocco et al., 2018). We have subsequently employed the MCG database to explore the investigation of these two drugs in clinical trials. The most frequent biomarkers in clinical trials investigating entrectinib were *ROS1* fusion, *NTRK1* fusion, and *NTRK2* fusion (Figure 4A). For larotrectinib, they were *NTRK1* fusion, *NTRK2* fusion, and *NTRK3* fusion (Figure 4B). Malignant solid tumors (n = 29), non-small cell lung carcinoma (n = 10), and colorectal carcinoma (n = 5) were the most common diseases studied in entrectinib clinical trials (Figure 4A), while malignant solid tumor (n = 21), non-Hodgkin lymphoma (n = 6), and congenital mesoblastic nephroma (n = 6) were the most prevalent diseases studied in larotrectinib clinical trials (Figure 4B).

Nine clinical trials studied larotrectinib, of which 9 were open and 0 were terminated. Of the trials that studied larotrectinib, 1 was early phase 1, 1 was phase 1/phase 2, and 7 were phase 2. Twelve clinical trials have studied entrectinib, 12 of which were open trials and 0 of which were closed trials. Of these trails, 1 was phase 1, 2 were phase 1/phase 2, 8 were phase 2, and 1 was phase 2/phase 3 (Figure 4C).

### 3.5 Adverse drug events (ADEs) related to entrectinib and larotrectinib in the FAERS database

**FIGURE 5:**
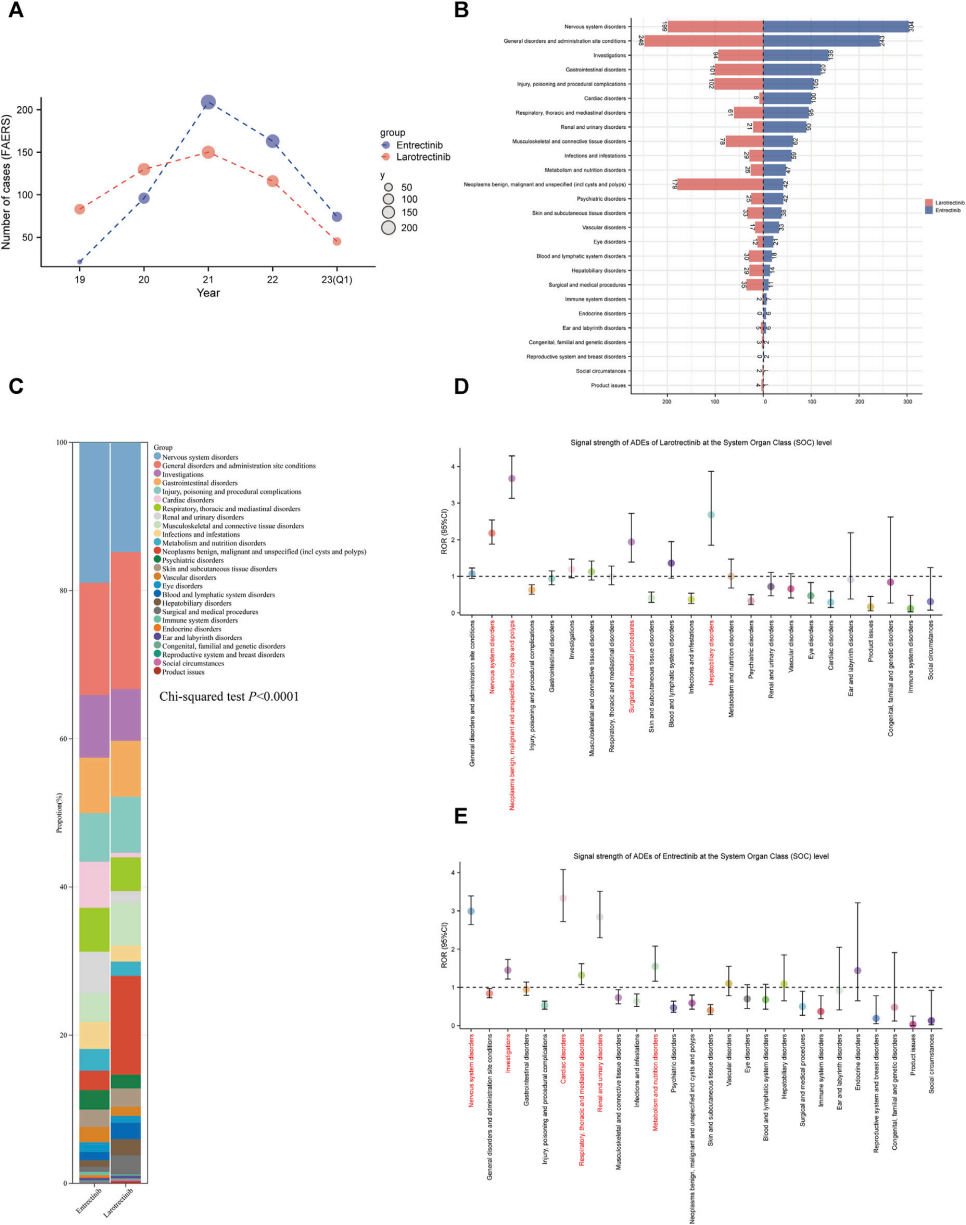
Signal detection at the SOC level. **(A)**. Annual distribution of ADE reports of the two first-generation *NTRK* inhibitors (entrectinib, blue; larotrectinib, red), from 2019 Q1 to 2023 Q1. **(B)**. Number of ADE reports for entrectinib and larotrectinib at the SOC level. **(C)**. The bar scale graph shows the percentage of ADEs at the SOC level. The ROR values and their corresponding 95% confidence intervals for larotrectinib **(D)** and entrectinib **(E)** are displayed for different levels of SOC. ADEs, adverse drug events; SOC, System Organ Class; Q1, first quarter; ROR, reporting odds ratio.

Subsequently, we conducted pharmacovigilance analyses in the FAERS database to investigate the signals of ADEs closely associated with the use of two first-generation *NTRK* inhibitors. In terms of the annual ADEs reported, entrectinib exhibited a steeper increase compared to larotrectinib, with both reaching their peaks in 2021. From that point, entrectinib continued to receive more ADE reports than larotrectinib (Figure 5A). Analysis of the baseline information for these ADEs revealed that the age group with the highest proportion of entrectinib users was below 18 years (5.7%), while for larotrectinib, it was the age group of 18–64 years (4.2%) (Table 2). However, it is important to note that approximately 90% of the reports had unknown age group information for both drugs. Regarding gender distribution, entrectinib had a higher proportion of female reports for ADEs (50.8%), whereas larotrectinib had a similar percentage of reports from males (42.0%) and females (42.4%). The top three countries contributing the highest number of reports were the United States (49.4%), Japan (24.7%), and Germany (2.5%) for entrectinib, and the United States (58.8%), France (7.3%), and Mexico (4.8%) for larotrectinib. In terms of report sources, health professionals accounted for the majority of reports for both drugs (65.5% for entrectinib and 73.3% for larotrectinib). While the primary indication for entrectinib was non-small cell lung carcinoma (29.7%), thyroid cancer (5.3%) was the main indication for larotrectinib. It is noteworthy that entrectinib (32.0%) had a higher proportion of reports of hospitalization compared to larotrectinib (22.6%) in cases of serious outcomes. However, both drugs exhibited similar proportions of reports for death, life-threatening events, and disability (Table 2).

**TABLE 2:** The demographic baseline data for adverse drug event (ADE) reports involving entrectinib and larotrectinib as the primary suspect (PS) drugs.

	Entrectinib			Larotrectinib	
Characteristics	Case number, n	Case proportion, %	Characteristics	Case number, n	Case proportion, %
Age (years)			Age (years)		
<18	32	5.7	<18	7	1.3
18–64	22	3.9	18–64	22	4.2
>64	2	0.3	>64	17	3.3
Unknown	507	90.1	Unknown	478	91.2
Gender			Gender		
Female	286	50.8	Female	222	42.4
Male	237	42.1	Male	220	42.0
Unknown	40	7.1	Unknown	82	15.6
Weight			Weight		
<80	163	29.0	<80	18	3.4
80–100	23	4.1	80–100	5	1.0
>100	13	2.3	>100	1	0.2
Unknown	364	64.6	Unknown	500	95.4
Reported Countries (top five)			Reported Countries (top five)		
US	278	49.4	US	308	58.8
JP	139	24.7	FR	38	7.3
DE	14	2.5	MX	25	4.8
IL	13	2.3	CA	18	3.4
FR	12	2.1	CH	15	2.9
Reported person			Reported person		
Consumer	180	32.0	Consumer	139	26.5
Health professional	369	65.5	Health professional	384	73.3
Unknown	14	2.5	Unknown	1	0.2
Indications (Top five)			Indications (Top five)		
Non-small cell lung cancer	167	29.7	Thyroid cancer	28	5.3
Lung neoplasm malignant	107	19.0	Neoplasm malignant	19	3.6
Neoplasm	37	6.6	Lung neoplasm malignant	18	3.4
Neoplasm malignant	23	4.1	Neoplasm	17	3.2
Lung adenocarcinoma	14	2.5	Sarcoma	14	2.7
Serious Outcomes			Serious Outcomes		
Other serious outcomes (OT)	229	44.2	Other serious outcomes (OT)	238	56.7
Hospitalization (HO)	166	32.0	Hospitalization (HO)	95	22.6
Death (DE)	103	19.9	Death (DE)	80	19.0
Life-threatening (LT)	14	2.7	Life-threatening (LT)	5	1.2
Disability (DS)	6	1.2	Disability (DS)	2	0.5

### 3.6 Signals detection at the System Organ Class (SOC) level

Figure 5B presented the reported numbers of the two *NTRK* inhibitors at the SOC level. For entrectinib and larotrectinib, ADEs were reported at 26 and 24 organ systems, respectively. Interestingly, no ADEs were reported for larotrectinib at the “endocrine disorder” and “reproductive system and breast disorders” SOCs (n = 0). In larotrectinib, the top three SOCs in terms of ADEs numbers were nervous system disorders (n = 304), general disorders and administration site conditions (n = 243), and investigations (n = 136). In entrectinib, they were, general disorders and administration site conditions (n = 248), nervous system disorders (n = 199), and neoplasms benign, malignant and unspecified (n = 179) (Figure 5B). Comparing the composition of each SOC between the two drugs, we found that entrectinib had a higher percentage of “nervous system disorders” (18.94% *versus* [vs.] 14.82%), “cardiac disorders” (6.23% vs. 0.60%), and “renal and urinary disorders” (5.61% vs. 1.56%) than larotrectinib, while “general disorders and administration site conditions” (18.47% vs. 15.14%) and “neoplasms benign, malignant and unspecified” (13.33% vs. 2.62%) were higher for larotrectinib. Statistical analyses also suggested that there was a significant difference in the proportion of SOCs between the two drugs (*p* < 0.0001, Figure 5C).

The disproportionality results of the three different algorithms at various SOC levels were shown in Table 3. Wherein, the number of SOCs meeting at least one algorithm signal value threshold was 4 and 6 for larotrectinib and entrectinib, respectively. For larotrectinib, they were nervous system disorders (SOC code:10029205, ROR 2.18 [1.88–2.54]), neoplasms benign, malignant and unspecified (SOC code:10029104, ROR 3.67 [3.13–4.29]), hepatobiliary disorders (SOC code:10019805, ROR 2.68 [1.85–3.87]), and surgical and medical procedures (SOC code:10042613, ROR 1.94 [1.39–2.72]). For entrectinib, they were nervous system disorders (SOC code:10029205, ROR 2.99 [2.64–3.39]), investigations (SOC code:10022891, ROR 1.45 [1.22–1.73]), cardiac disorders (SOC code:10007541, ROR 3.33 [2.72–4.08]), respiratory, thoracic and mediastinal disorders (SOC code:10038738, ROR 1.32 [1.07–1.62]), renal and urinary disorders (SOC code:10038359, ROR 2.84 [2.30–3.51]), and metabolism and nutrition disorders (SOC code:10027433, ROR 1.55 [1.16–2.08]). To improve visualization, Figures 5D,E depicted the signal strength (ROR, with 95% confidence interval [CI]) at the SOC level for larotrectinib and entrectinib, respectively.

**TABLE 3:** Signal detection at the SOC level. Lower right marker 1, entrectinib vs all other drugs; Lower right marker 2, larotrectinib vs all other drugs. ROR, reporting odds ratio; CI, confidence interval; PRR, proportional reporting ratio; χ2, chi-squared; IC, information component; IC025, the lower limit of the 95% CI of IC; SOC, System Organ Class.

SOC name	Case number1	Case number2	ROR1 (95 CI%)	ROR2 (95 CI%)	PRR1	PRR2	χ21	χ22	ICO251	ICO252
Nervous system disorders	304	199	2.99 (2.64–3.39)	2.18 (1.88–2.54)	2.61	2.01	326.03	108.46	−0.28	−0.66
General disorders and administration site conditions	243	248	0.84 (0.73–0.97)	1.07 (0.94–1.23)	0.87	1.06	6.12	1.03	−1.88	−1.58
Investigations	136	94	1.45 (1.22–1.73)	1.19 (0.96–1.47)	1.41	1.17	17.43	2.60	−1.17	−1.44
Gastrointestinal disorders	120	101	0.95 (0.79–1.14)	0.94 (0.77–1.15)	0.95	0.94	0.32	0.38	−1.74	−1.75
Injury, poisoning and procedural complications	105	102	0.53 (0.43–0.64)	0.63 (0.51–0.77)	0.56	0.65	41.83	21.04	−2.51	−2.28
Cardiac disorders	100	8	3.33 (2.72–4.08)	0.29 (0.15–0.59)	3.18	0.30	152.70	13.48	0.01	−3.41
Respiratory, thoracic and mediastinal disorders	95	61	1.32 (1.07–1.62)	0.99 (0.77–1.28)	1.30	0.99	6.77	0	−1.29	−1.68
Renal and urinary disorders	90	21	2.84 (2.30–3.51)	0.72 (0.47–1.11)	2.74	0.73	101.26	2.20	−0.22	−2.13
Musculoskeletal and connective tissue disorders	62	78	0.73 (0.57–0.94)	1.13 (0.90–1.42)	0.74	1.12	6.03	1.11	−2.10	−1.50
Infections and infestations	59	29	0.64 (0.50–0.83)	0.37 (0.26–0.54)	0.66	0.39	11.22	29.68	−2.27	−3.03
Metabolism and nutrition disorders	47	26	1.55 (1.16–2.08)	1.00 (0.68–1.47)	1.54	1.00	9.02	0	−1.05	−1.67
Psychiatric disorders	42	25	0.47 (0.35–0.64)	0.33 (0.23–0.50)	0.49	0.35	24.18	32.39	−2.71	−3.19
Neoplasms benign, malignant and unspecified (incl cysts and polyps)	42	179	0.59 (0.43–0.80)	3.67 (3.13–4.29)	0.60	3.31	11.94	300.84	−2.41	0.06
Skin and subcutaneous tissue disorders	38	33	0.40 (0.29–0.55)	0.41 (0.29–0.57)	0.41	0.42	33.70	28.10	−2.94	−2.92
Vascular disorders	33	17	1.10 (0.78–1.55)	0.66 (0.41–1.07)	1.09	0.67	0.27	2.88	−1.54	−2.25
Eye disorders	21	12	0.70 (0.45–1.07)	0.47 (0.27–0.83)	0.70	0.48	2.70	7.02	−2.18	−2.74
Blood and lymphatic system disorders	18	30	0.68 (0.43–1.08)	1.36 (0.95–1.95)	0.68	1.35	2.70	2.80	−2.22	−1.23
Hepatobiliary disorders	14	29	1.09 (0.65–1.85)	2.68 (1.85–3.87)	1.09	2.64	0.11	29.83	−1.54	−0.27
Surgical and medical procedures	11	35	0.50 (0.27–0.90)	1.94 (1.39–2.72)	0.50	1.92	5.59	15.55	−2.67	−0.73
Immune system disorders	7	2	0.37 (0.18–0.78)	0.12 (0.03–0.48)	0.38	0.12	7.38	12.92	−3.08	−4.71
Ear and labyrinth disorders	6	5	0.92 (0.41–2.05)	0.91 (0.38–2.19)	0.92	0.91	0.04	0.05	−1.79	−1.81
Endocrine disorders	6	0	1.44 (0.65–3.21)	NA	1.44	NA	0.80	NA	−1.14	NA
Reproductive system and breast disorders	2	0	0.19 (0.05–0.78)	NA	0.20	NA	6.64	NA	−4.02	NA
Congenital, familial and genetic disorders	2	3	0.48 (0.12–1.91)	0.84 (0.27–2.62)	0.48	0.84	1.14	0.09	−2.73	−1.91
Product issues	1	4	0.03 (0.00–0.25)	0.17 (0.06–0.45)	0.04	0.17	26.60	16.32	−6.48	−4.21
Social circumstances	1	2	0.13 (0.02–0.92)	0.31 (0.08–1.24)	0.13	0.31	5.82	3.08	−4.61	−3.35

### 3.7 Differences in ADEs of entrectinib and larotrectinib at the preferred term (PT) level

**FIGURE 6:**
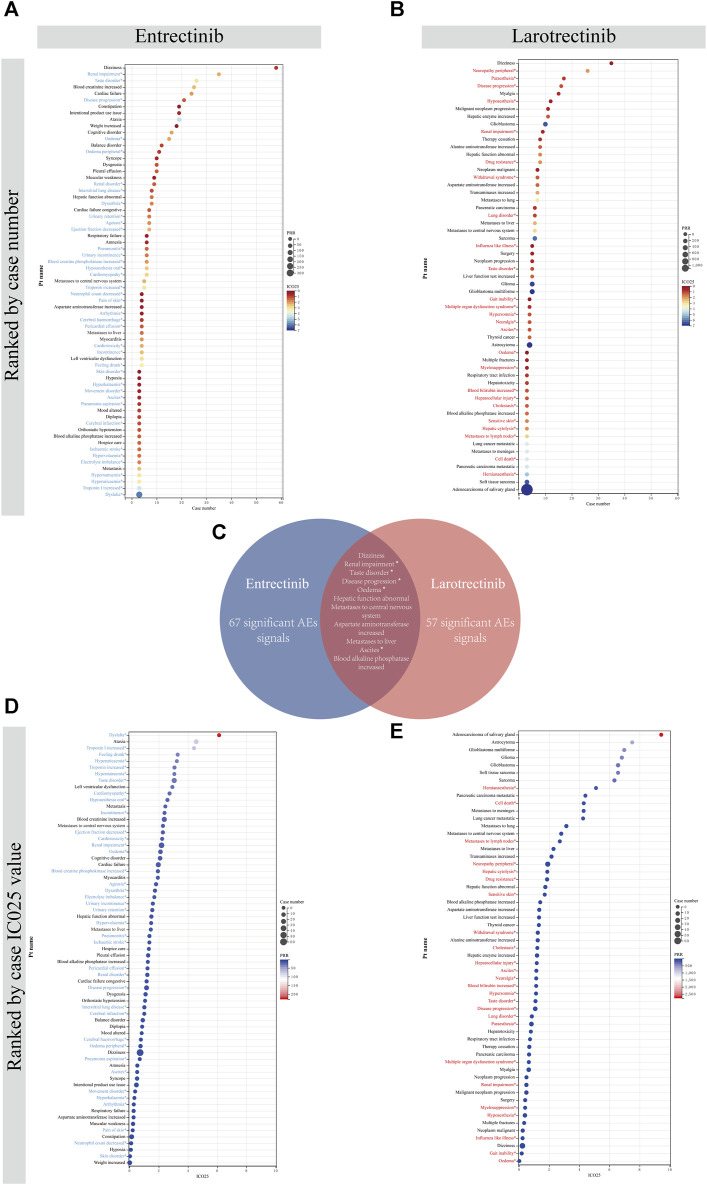
Comparison of signal values of the two first-generation *NTRK* inhibitors for ADEs at the PT level. All positive signals for entrectinib **(A)** and larotrectinib **(B)** are ranked by case number. **(C)**. Intersection of positive signals of two drugs. All positive signals for entrectinib **(D)** and larotrectinib **(E)** are ranked by IC025 value. An asterisk indicates that the signal is not indicated in the drug instruction. PRR, proportional reporting ratio; PT, preferred term; IC, information component; IC025, the lower limit of the 95% confidence interval of IC.

After satisfying the thresholds of the three algorithms simultaneously, we detected 67 entrectinib-related ADEs and 57 larotrectinib-related ADEs at the PT level, respectively. We sorted these screened ADEs by case number and IC025 value, respectively. For entrectinib, signals including dizziness (n = 58), renal impairment (n = 35), and taste disorder (n = 26) had the highest reported cases, which suggested that they were more common in reports of ADEs following entrectinib dosing (Figure 6A). Dysplasia (6.13), ataxia (4.57), and troponin I increased (4.14) had the highest IC025 values, indicating that they were more associated with entrectinib dosing than the other drugs (Figure 6D). For larotrectinib, dizziness (n = 35), neuropathy peripheral (n = 26), and paraesthesia (n = 17) had the largest number of reports (Figure 6B), while adenocarcinoma of salivary gland (9.43), astrocytoma (7.51), and glioblastoma multiforme (6.98) had the highest IC025 values (Figure 6E).

Besides, a large number of unexpected signals were identified and highlighted in prominent colors. These signals need to be refined in subsequent updates of the drug instruction. After taking the intersection of ADEs for both drugs, we found a total of 11 overlapping signals (Figure 6C). Of them, renal impairment, taste disorder, disease progression, oedema, and ascites were intersected unexpected signals, which needed to be given sufficient attention in subsequent clinical studies. All signals and calculations that satisfied the thresholds of the three algorithms were listed in Supplementary Table S5.

### 3.8 Time to onset (TTO) analysis of all ADEs

**FIGURE 7:**
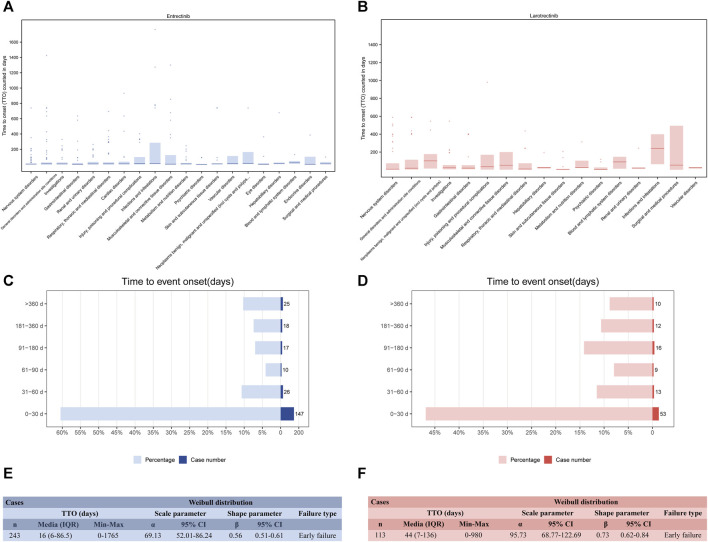
Time to onset (TTO) analysis (counted in days). Box plot of the TTO at the SOC level for entrectinib **(A)** and larotrectinib **(B)**. Bold bar within the stick: median TTO; Lower end of the stick: 1/4 quantile of the TTO; Upper end of the stick: 3/4 quantile of the TTO. Number and proportion of all TTO reports in different time periods for entrectinib **(C)** and larotrectinib **(D)**. Weibull distribution test of TTO for entrectinib **(E)** and larotrectinib **(F)**. A TTO of 0 days signifies that the adverse event occurred on the same day as the start of treatment. IQR, interquartile range.

Recognizing the onset time of these ADEs can enable healthcare professionals better target their post-medication monitoring. After data filtering (removing missing and incorrect reporting times), 243 and 113 TTO reports were collected related to entrectinib and larotrectinib, respectively. The median TTO for entrectinib and larotrectinib was 16 days (Figure 7E) and 44 days (Figure 7F). Observing these TTO at the SOC level, we noticed that the SOCs with longer onset after entrectinib administration included “blood and lymphatic system disorders” (median TTO: 23 days), and “neoplasms benign, malignant and unspecified” (median TTO: 17 days), while “psychiatric disorders” (median TTO: 1 day), “eye disorders” (median TTO: 4 days), and “gastrointestinal disorders” (median TTO: 4 days) had shorter onset (Figure 7A). For larotrectinib, “infections and infestations” (median TTO: 242 days) and “neoplasms benign, malignant and unspecified” (median TTO: 101 days) had longer onset time, while “psychiatric disorders” (median TTO: 4 days), “skin and subcutaneous tissue disorders” (median TTO: 8 days), and “nervous system disorders” (median TTO: 9 days) had shorter onset time (Figure 7B). The specific statistical description of TTO was in Supplementary Table S6.

Concerning the distribution of ADEs over time, Figures 7C,D indicated that the majority of ADEs occurred within the first month after *NTRK* inhibitors use (n = 147, 60.5% for entrectinib; n = 53, 46.9% for larotrectinib). As time was delayed, the number of ADEs decreased but leveled off. Of note, our data showed that ADEs could still occur after 1 year of drug administration (n = 25, 10.3% for entrectinib; n = 10, 8.9% for larotrectinib) (Supplementary Table S6). The Weibull shape parameter analysis revealed calculated shape parameters β) of 0.56 (0.51–0.61) for entrectinib (Figure 7E) and 0.73 (0.62–0.84) for larotrectinib (Figure 7F), both with β values <1, indicating an early failure type. This suggested that for both *NTRK* inhibitors, the incidence of ADE decreased over time.

## 4 Discussion

Precision medicine endeavors to develop medical interventions personalized to an individual’s genetic, environmental, and lifestyle factors (Wahida et al., 2023). In recent years, with the rapid advancement of precision medicine, there has been a shift in clinical trials from being focused on specific tumor types to being oriented towards the genome and agnostic to histology (Fountzilas et al., 2022). In this broader context, genomics offers valuable insights into identifying dysregulated genes, associated pathways, and histological knowledge within complex disease pathology. Clinical trials play a crucial role in translating genomic evidence into effective drug treatments, bridging the gap between bench research and bedside application (Knoepfler, 2015). Pharmacovigilance, as a critical component of drug safety regulation, is essential in identifying ADEs that occur during clinical trials and post-marketing, ensuring medication safety. Conversely, analyzing ADEs in isolation without understanding the underlying molecular etiology of the observed signals can lead to “fragmentation” in the relationship between ADEs and genomic alterations. By integrating genomic-level evidence and participant target data from clinical trials, this fragmentation can be partially mitigated, aiding in the identification of high-risk individuals and populations for ADEs, thus enhancing drug safety, efficacy, and overall public health (Zhou et al., 2023). Recently, studies have emerged linking genomics and pharmacovigilance. For instance, Jing et al. assessed the relationship between multiple genomic factors and immune-associated adverse events in various cancer types by combining genomic data with pharmacovigilance data (Jing et al., 2020). In our study, we conducted a comprehensive mapping of the *NTRK* genome, utilizing multi-omics data from TCGA, followed by a review of clinical trials involving *NTRK* inhibitors and an extensive pharmacovigilance analysis using the FAERS database. Our genomic evidence provides valuable insights into mitigating adverse effects of first-generation *NTRK* inhibitors, such as drug resistance, while pharmacovigilance analyses offer crucial additions by uncovering undetected adverse effects in clinical trials and aiding in optimal drug design at the genomic level.

### 4.1 *NTRK* genes expression and genomic alteration

After analyzing the expression profiles of *NTRK* genes, we observed that the mRNA expression of *NTRK1/2/3* was generally downregulated in tumor tissues compared to normal tissues across most cancers. Additionally, we identified a significant negative correlation between the hypermethylation state of the *NTRK* gene promoters and their mRNA expression in multiple cancer types. Previous studies have also reported aberrant methylation patterns in the *NTRK* genes in various cancers. For instance, Yamada et al. demonstrated elevated methylation levels in PRAD cell lines for *NTRK*2, which resulted in decreased mRNA expression (Yamada et al., 2004). Similarly, hypermethylation status of *NTRK*3 was reported in cervical cancer through analyzing sequencing data and further validated in clinical samples (Ji et al., 2021). Considering that epigenetic changes are an essential mechanism for regulating gene expression, aberrant DNA methylation might be a plausible explanation for the dysregulation of *NTRK* gene expression (Arechederra et al., 2018). Moreover, CNV of DNA sequences can remodel gene structure, regulate gene expression, and facilitate significant phenotypic variation, including *NTRK* genes (Zhou et al., 2011; Tsai et al., 2016; Yu et al., 2021). Our study also uncovered the presence of CNVs in *NTRK* genes in many cancers, particularly in UCS and LUAD. This observation suggests that CNVs might influence the mRNA expression and function of these genes. However, further experiments are needed to validate this hypothesis, as there is limited evidence available from previous studies. Additionally, we observed a protective role of *NTRK3* in BRCA and PRAD, as well as a protective role of *NTRK2* in LGG. These findings are consistent with previous research and suggest that these genes may exert a protective effect through mechanisms such as immune cell recruitment, angiogenesis inhibition, and apoptosis induction (Bouzas-Rodriguez et al., 2010; Luo et al., 2013; Bagherabadi et al., 2022; Fang et al., 2023). However, we also noticed that high *NTRK* expression was hazardous for patients with BLCA and UVM. The varying prognoses in different types of tumors may be attributed to the fact that the function of *NTRK* genes is receptor-dependent. In the presence of a ligand, these genes transmit positive, proliferative signals, whereas they induce apoptosis in the absence of a ligand (Tauszig-Delamasure et al., 2007; Luo et al., 2013). Also, the effect of tumor heterogeneity and different splicing patterns on its function cannot be overlooked (Bagherabadi et al., 2022).

In our further exploration of the genomic alterations of *NTRK* genes, we discovered that in pan-cancer, alterations in these genes were predominantly characterized by high mRNA expression, amplifications, and mutations. The most frequently mutated amino acid sites in *NTRK1/2/3* were G169R, A268V, and R153L/Q, respectively. Prior research has indicated that non-fusion *NTRK* alterations, such as mutations and amplifications, are associated with the response to *NTRK* inhibitors and their therapeutic efficacy (Okamura et al., 2018; Amatu et al., 2019; Zito Marino et al., 2023). In particular, mutations at the G595R and G667C sites of *NTRK1*, and at G696A and G623R of *NTRK3*, have been associated with the acquisition of resistance to first-generation *NTRK* inhibitors, possibly due to the fact that these point mutations alter the three-dimensional conformation of the kinase’s structural domains, thereby reducing or abolishing binding to the inhibitors (Russo et al., 2016; Drilon et al., 2017; Somwar et al., 2020). In contrast, in a case report by Hempel et al., a patient with ESCA with *NTRK1* gene amplification experienced shrinkage of primary and metastatic tumors within 6 weeks after treatment with the *NTRK* inhibitor larotrectinib, accompanied by a decrease in tumor markers (Hempel et al., 2020). Finally, we also explored potential signaling pathways for genes co-altered with *NTRK* genes, which included PI3K/Akt, cGMP-PKG, and cAMP, etc. Mutational pathways co-activated with target genes are frequently observed in targeted therapies (Moore et al., 2020). For example, resistance to BRAF inhibitors is linked to the activation of the MAPK pathway, necessitating the co-administration of MEK inhibitors under certain circumstances (Yi et al., 2022). Likewise, combining *NTRK* inhibitors with co-mutational pathway inhibitors (such as PI3K inhibitors) could represent a potential therapeutic strategy for patients resistant to first-generation *NTRK* inhibitors. In conclusion, our study offers a comprehensive understanding of *NTRK* genomic alterations. While non-fusion alterations have not yet demonstrated consistent and long-lasting response to targeted therapies, they may still yield valuable insights into mechanisms of drug resistance.

### 4.2 Fusion mutations of *NTRK* genes

Structural rearrangements of the genome can lead to the formation of fusion genes, which possess oncogenic properties and subsequently result in the overexpression of aberrant proteins. These abnormal proteins are known to drive tumorigenesis and present potential targets for therapeutic intervention (Mitelman et al., 2007; Bastus et al., 2010; Best et al., 2018). While *NTRK* gene fusions occur at a low frequency in common cancer types like lung and colorectal cancers (<25%), they are observed at a high frequency in specific rare tumor types (>80%). These fusions activate downstream cell growth and proliferative pathways, thereby promoting tumorigenesis (Vaishnavi et al., 2015; Amatu et al., 2016; Amatu et al., 2019). Moreover, constitutively active TRK kinase generated by gene fusion becomes a target for the action of *NTRK* inhibitors (Wang et al., 2015; Khotskaya et al., 2017). Our study provided a comprehensive analysis of fusion mutations involving *NTRK* genes across various cancer types. We observed that the majority of these fusion mutations occurred in *NTRK3*, with THCA having the highest reported incidence. Among the fusion pairs, *ETV6-NTRK3* was found to have the highest percentage. Furthermore, most of these fusion mutations were in-frame mutations. Consistent with our findings, research studies have increasingly demonstrated the presence of the *ETV6-NTRK3* fusion in various tumor types, including glioblastoma, ductal carcinoma, fibrosarcoma, and THCA (Tognon et al., 2002; Bastos et al., 2018; Biswas et al., 2022; Jiang, 2022). Interestingly, the study by Kinnunen et al. employed proximity-labeled mass spectrometry to establish a stable, reliable association of the *ETV6-NTRK3* fusion with several key signaling pathways, including ERBB, IRS-1, and JAK/STAT (Kinnunen et al., 2023). As another of the more common and first reported *NTRK* fusion mutations, *TPM3-NTRK1* was initially identified in colorectal cancer (CRC) samples, and subsequent studies have confirmed that chromosomal rearrangements in this manner make patients with CRC highly sensitive to TRKA inhibitors (Martin-Zanca et al., 1986; Ardini et al., 2014; Créancier et al., 2015). Other fusion types, such as *SQSTM1-NTRK1*, although carried by only one patient with THCA in our study, a recent case report demonstrated that patients carrying *SQSTM1-NTRK1* had a 51% reduction in tumor burden after 18 months of treatment with larotrectinib, and this impressive efficacy continued (Bargas et al., 2022). Of course, we have identified other novel fusion types including *SQSTM1-NTRK2*, *FAT1-NTRK3*, and *AKAP13-NTRK3*, however the relevant reports are very limited so far. Whether they could become new targets for *NTRK* inhibitors needs to be validated in subsequent clinical trials (Bargas et al., 2022).

### 4.3 Detection of adverse drug event

Genomic evidence indicates that both larotrectinib and entrectinib are pan-TRK inhibitors, targeting TRKA, TRKB, and TRKC. However, it is important to note that these TRK receptors also play essential roles in neurodevelopment. Consequently, the use of these pan-TRK inhibitors may lead to treatment-related side effects due to the inhibition of TRK signaling in normal tissues (Bhangoo and Sigal, 2019). Our pharmacovigilance study revealed that entrectinib had positive signaling values at six SOC levels compared to four for larotrectinib. Of particular note, both entrectinib (ROR: 2.99 [2.64–3.39]) and larotrectinib (ROR: 2.18 [1.88–2.54]) had positive signal values at the “nervous system disorders”. Generally, neurological ADEs are primarily thought to be related to the on-target effects of TRK inhibitors (Kummar et al., 2023). Considering the critical role of *NTRK1* and *NTRK3* in the normal functioning of sensory neurons, their loss-of-function mutations may lead to dizziness, sensory abnormalities, headaches and gait disturbances (Cocco et al., 2018; Amatu et al., 2019). Another anticipated ADE is weight gain, due to the vital role of *NTRK2* in controlling energy balance and appetite (An et al., 2020; Houtz et al., 2021) Consistently, at the PT level, we also identified several common, drug-related on-target adverse reactions, including dizziness, constipation, ataxia, weight increased, balance disorder, and dysgeusia. These ADEs we identified are generally in line with previously reported adverse events in clinical trials of entrectinib. Although the majority of these treatment-related adverse events (TRAEs) were grade 1/2, weight gain (5%–10%) was considered the most common grade 3/4 event, and central nervous system toxicity was reported as the most severe TRAE (3%–4%) (Al-Salama and Keam, 2019; Doebele et al., 2020; Drilon et al., 2020; Dziadziuszko et al., 2021). Therefore, it is crucial to implement timely monitoring and provide appropriate guidance to patients. However, it is interesting to note that entrectinib has shown a potential protective effect in patients with tumors that may have a higher risk of central nervous system metastasis. This could be attributed to its ability to more easily penetrate the blood-brain barrier (Dziadziuszko et al., 2021; Frampton, 2021). How to balance adverse effects and potential benefits to optimize drug selection in the future also needs to be explored in depth.

Although previous clinical trials have shown similar safety profiles for both drugs, in our study we demonstrated that entrectinib had stronger signal values for “cardiac disorders”, “respiratory, thoracic and mediastinal disorders”, “renal and urinary disorders” and “metabolism and nutrition disorders”, while larotrectinib had stronger signal values for “neoplasms benign, malignant and unspecified”, and “hepatobiliary disorders”. For entrectinib, cardiac problem is the other most common type of severe TRAE (Drilon et al., 2020; Marcus et al., 2021). In a case of NSCLC with *ROS1* rearrangement, the patient developed drug-induced heart failure after treatment with entrectinib, and the symptoms improved after drug discontinuation (Otsu et al., 2022). Results from another previous pharmacovigilance analysis also revealed that *ALK* and *ROS1* inhibitors induced higher odds of cardiac conduction disease than other targeted therapies (Waliany et al., 2021). Moreover, at the PT level, we also detected positive signal values including “interstitial lung disease”, “respiratory failure”, and “pneumonitis” with a relatively high number of cases at the respiratory level, which may be due to the rich blood supply of the lungs, causing accumulation of the drug, or the release of toxic substances (Long and Suresh, 2020; Spagnolo et al., 2022). Elevated aminotransferase occurred in half of the adverse event reports during the clinical trials of larotrectinib and was also a primary cause of drug dose reductions (Drilon et al., 2018; Laetsch et al., 2018; Hong et al., 2020; Le et al., 2022). In light of these findings, it is important to conduct regular and dynamic monitoring of the patient’s liver function and to administer suitable hepatoprotective agents. Additionally, we observed several ADEs related to neoplasm progression for larotrectinib, including “malignant neoplasm progression,” “neoplasm malignant,” and “metastases to lung”. These adverse signals may be more associated with disease progression rather than being solely attributed to adverse reactions caused by larotrectinib. This is supported by the fact that a significant majority of patients experienced objective remission following treatment with the larotrectinib (Doebele et al., 2020; Doz et al., 2022; Drilon et al., 2022). In conclusion, our analysis has provided a comparative evaluation of the signal strength in different organs for both larotrectinib and entrectinib. This comprehensive assessment can contribute to a more detailed and proactive clinical medication monitoring approach, enhancing patient safety and optimizing the effectiveness of treatment.

### 4.4 Unexpected signals

Furthermore, our study revealed some unexpected signals. For entrectinib, we observed unexpected signals with a high number of cases, such as taste disorder and renal impairment. Furthermore, we identified unexpected signals with a low number of cases but a high signal intensity, including dyslalia and hyperuricemia. It is worth noting that taste disorder and dyslalia have been hypothesized to be a result of the potential on-target effects of *NTRK* inhibitors (Naik et al., 2010; Ma et al., 2011; Szobota et al., 2019). Hyperuricemia has been recognized in previous clinical trials as a serious toxicity induced by entrectinib treatment that required intervention, but the exact cause is unknown (Dziadziuszko et al., 2021; Marcus et al., 2021). Similarly, regarding larotrectinib, there were more case reports of neuropathy peripheral, paresthesia, and stronger signal strength for hemianesthesia, and drug-resistance. Regarding the mechanism of resistance to larotrectinib, in addition to being associated with mutations or altered pathway activation as described above, it may also relate to the mode of administration (e.g., intermittent dosing), which may require a combination of other drugs to overcome (Amatu et al., 2016; Cocco et al., 2019; Kummar et al., 2023). Remarkably, unexpected signals found in both drugs included, renal impairment, taste disorder, disease progression, edema, and ascites. Some previous clinical trials failed to detect the new signals, whereas our results complement the previous studies and are indicative of subsequent updates of drug instruction (Doebele et al., 2020; Hong et al., 2020; Doz et al., 2022). Furthermore, as both *NTRK* inhibitors are predominantly metabolized by the enzyme cytochrome P450 (CYP450), which has been strongly linked to hepatic and renal drug toxicity, investigating CYP450 site variation at the pharmacogenomic level could yield distinctive insights into the toxicity of *NTRK* inhibitors (Quintanilha et al., 2017; Wang et al., 2020).

It is important to note that while we have made certain genomic insights regarding the adverse effects (e.g., drug resistance) of first-generation *NTRK* inhibitors, more reliable conclusions about causality may require higher-level genetic evidence in the future. This could involve identifying single nucleotide polymorphism (SNP) loci significantly associated with ADEs through genome-wide association studies (Ghouse et al., 2022; Giles et al., 2022). Such an approach could enable the screening and identification of patients susceptible to adverse effects, thereby improving efficacy and concurrently mitigating adverse effects.

### 4.5 Time to onset analysis

Some ADEs can occur shortly after starting treatment, ranging from minutes to hours. However, other ADEs may manifest days, weeks, months, or even years after exposure. The timing of these events can vary depending on various factors, including the drug’s pharmacokinetics and its metabolites, as well as the underlying pathophysiological mechanism of action (Leroy et al., 2014). Previous studies have provided information on various ADEs, but the precise timing of these events remains largely unknown. In our study, we found that both drugs exhibited earlier onset of ADEs in the SOC categories of “psychiatric disorders” and “nervous system disorders,” while ADEs related to “infections and infestations” and “neoplasms benign, malignant, and unspecified” occurred later. However, there were notable differences between the two drugs. For instance, ADEs in the category of “musculoskeletal and connective tissue disorders” had a median time to onset of 6 days in entrectinib and 53 days in larotrectinib. In both drugs, these ADEs predominantly occurred within the first month of administration, with a progressive decrease in their probability of occurrence over time. This highlights the significance of early medication surveillance. Overall, our analysis provided valuable insights into the onset timing of ADEs for two *NTRK* inhibitors, facilitating a more meticulous approach to preventing or diagnosing the occurrence of ADEs.

Our study exhibits several strengths. Firstly, the comprehensive analysis of data from multiple sources contributes to a more thorough understanding of the *NTRK* genes. Secondly, the precise application of multiple analysis methods and the depth of the results further underscore the strength of this study. However, it is crucial to acknowledge certain limitations. Firstly, the small sample sizes for specific cancer types may introduce bias into the analyses concerning these types of cancer. Moreover, the FAERS database does not establish a causal relationship between drug use and ADEs, and limited drug dosage information hindered our analysis of the correlation between ADEs and drug dosage. Lastly, due to the recent introduction of the two *NTRK* inhibitors to the market, reported ADEs were limited, necessitating further validation of our results with larger sample sizes in future studies.

## 5 Conclusion

This study delineated the genomic features of *NTRK*, encompassing expression, methylation, and gene fusion, through the analysis of multi-omics data. Subsequently, comprehensive analyses were conducted to compare the safety profiles of two first-generation *NTRK* inhibitors using the FAERS database. These analyses offer valuable insights for healthcare professionals, aiding their understanding of the mechanisms of resistance to *NTRK* inhibitors and facilitating the monitoring of adverse effects associated with entrectinib and larotrectinib.

## Data Availability

The original contributions presented in the study are included in the article/Supplementary Material, further inquiries can be directed to the corresponding author.
