# Supplementary material for: From genomic spectrum of NTRK genes to adverse effects of its inhibitors, a comprehensive genome-based and real-world pharmacovigilance analysis
Source: Front Pharmacol. 2024 Jan 31;15:1329409. doi: 10.3389/fphar.2024.1329409 (PMC10864613; doi:10.3389/fphar.2024.1329409)
Supplement: Supplementary file 3 [file Table1.docx]

**Abbreviation:**

ACC: adrenocortical carcinoma

BLCA: bladder urothelial carcinoma

BRCA: breast invasive carcinoma

CESC: cervical squamous cell carcinoma and endocervical adenocarcinoma

CHOL: cholangiocarcinoma

COAD: colon carcinoma

DLBC: lymphoid neoplasm diffuse large B-cell lymphoma

ESCA: esophageal carcinoma

GBM: glioblastoma multiforme

HNSC: Head and Neck squamous cell carcinoma

KICH: kidney chromophobe

KIRC: kidney renal clear cell carcinoma

KIRP: kidney renal papillary cell carcinoma

LAML: acute myeloid leukemia

LGG: brain lower grade glioma

LIHC: liver hepatocellular carcinoma

LUAD: lung adenocarcinoma

LUSC: lung squamous cell carcinoma

MESO: mesothelioma

OV: ovarian serous cystadenocarcinoma

OSCC: oral squamous cell carcinoma

PAAD: pancreatic adenocarcinoma

PCPG: pheochromocytoma and paraganglioma

PRAD: prostate adenocarcinoma

READ: rectum adenocarcinoma

SARC: sarcoma

SKCM: skin cutaneous melanoma

STAD: stomach adenocarcinoma

TGCT: testicular germ cell tumors

THCA: thyroid carcinoma

THYM: thymoma

UCEC: uterine corpus endometrial carcinoma

UCS: uterine carcinosarcoma

UVM: uveal melanoma
