# Supplementary figures and images for: From genomic spectrum of NTRK genes to adverse effects of its inhibitors, a comprehensive genome-based and real-world pharmacovigilance analysis
Source: Front Pharmacol. 2024 Jan 31;15:1329409. doi: 10.3389/fphar.2024.1329409 (PMC10864613; doi:10.3389/fphar.2024.1329409)

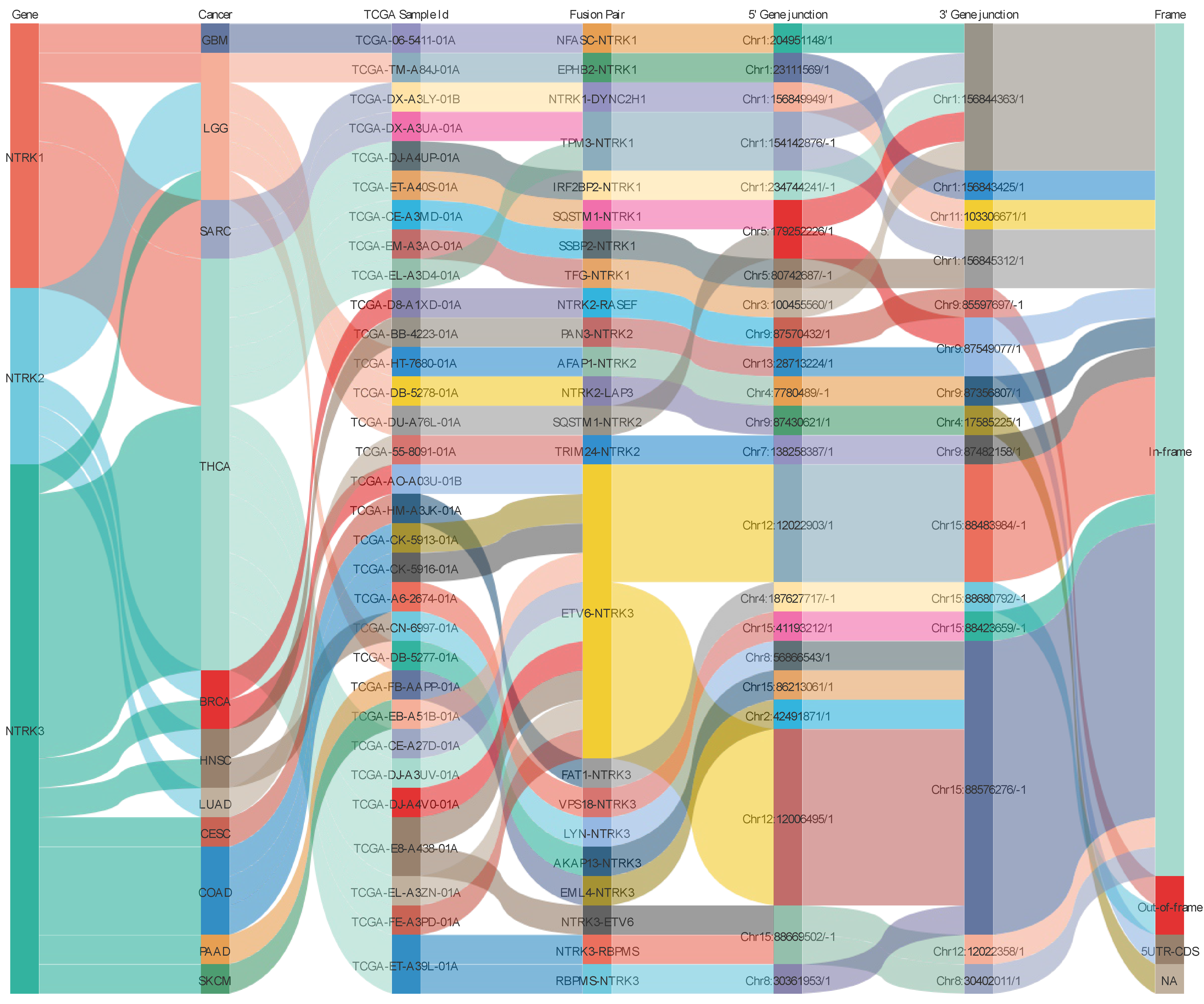

Supplement: Supplementary file 4 [file Image2.tif]

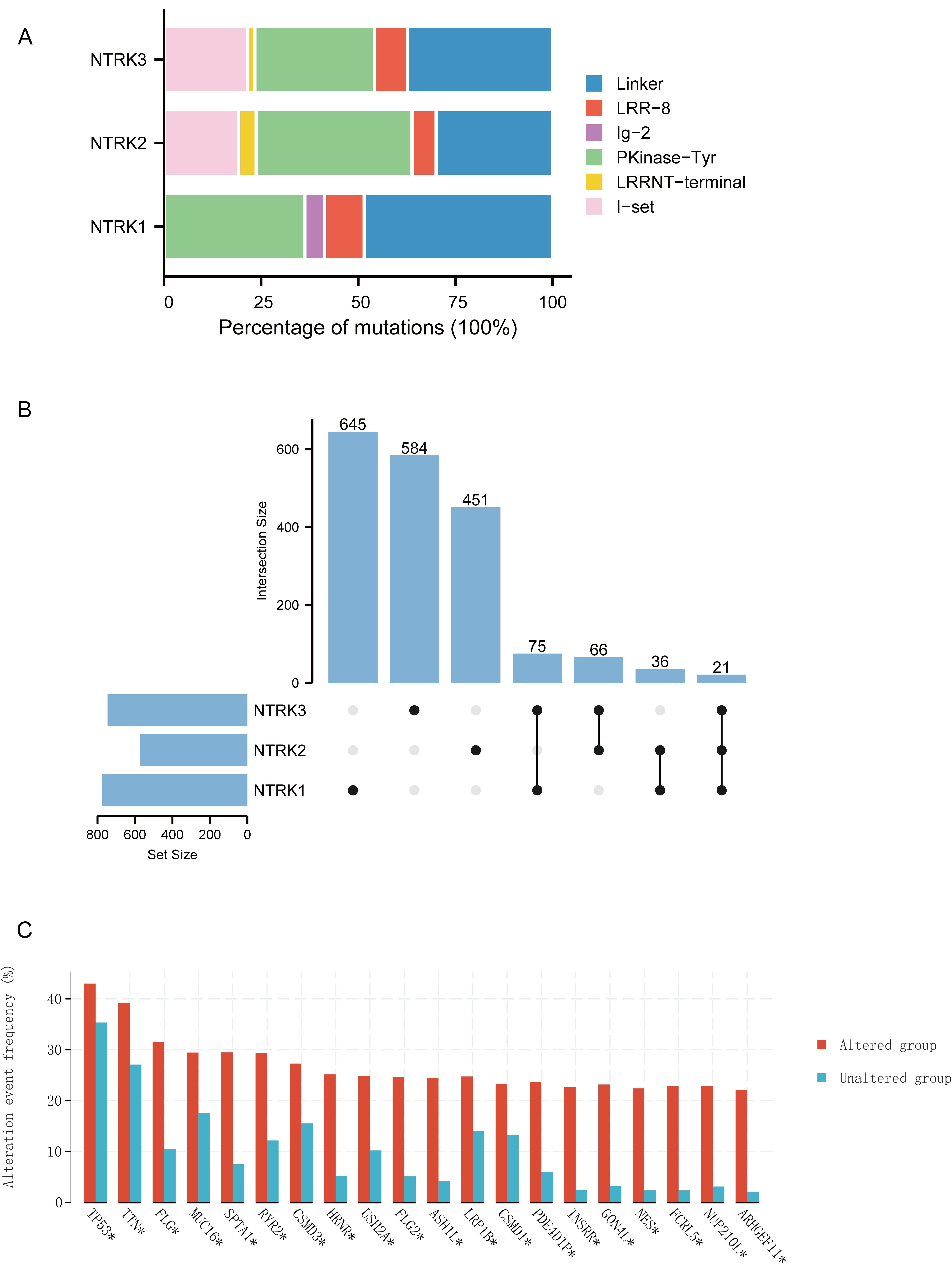

Supplement: Supplementary file 5 [file Image1.tif]
