# Supplementary material for: From genomic spectrum of NTRK genes to adverse effects of its inhibitors, a comprehensive genome-based and real-world pharmacovigilance analysis
Source: Front Pharmacol. 2024 Jan 31;15:1329409. doi: 10.3389/fphar.2024.1329409 (PMC10864613; doi:10.3389/fphar.2024.1329409)
Supplement: Supplementary file 6 [file Table5.docx]

Entrectinib Larotrectinib

# SOC name

Nervous system disorders Renal and urinary disorders Nervous system disorders Investigations

Cardiac disorders

General disorders and administration site conditions Gastrointestinal disorders

Injury, poisoning and procedural complications Nervous system disorders

Investigations

Nervous system disorders

General disorders and administration site conditions Nervous system disorders

General disorders and administration site conditions Nervous system disorders

Nervous system disorders

Respiratory, thoracic and mediastinal disorders Musculoskeletal and connective tissue disorders Renal and urinary disorders

Respiratory, thoracic and mediastinal disorders Hepatobiliary disorders

Nervous system disorders Cardiac disorders

Renal and urinary disorders Nervous system disorders Investigations

Respiratory, thoracic and mediastinal disorders Nervous system disorders

Respiratory, thoracic and mediastinal disorders Renal and urinary disorders

Investigations Gastrointestinal disorders Cardiac disorders

Neoplasms benign, malignant and unspecified (incl cysts and polyps) Investigations

Investigations

Skin and subcutaneous tissue disorders Investigations

Cardiac disorders Nervous system disorders Cardiac disorders

Neoplasms benign, malignant and unspecified (incl cysts and polyps) Cardiac disorders

Cardiac disorders

Renal and urinary disorders Cardiac disorders

General disorders and administration site conditions Skin and subcutaneous tissue disorders

Respiratory, thoracic and mediastinal disorders Metabolism and nutrition disorders

Nervous system disorders Gastrointestinal disorders Infections and infestations Psychiatric disorders

Eye disorders

Nervous system disorders Vascular disorders Investigations

Surgical and medical procedures Nervous system disorders Metabolism and nutrition disorders Metabolism and nutrition disorders

Neoplasms benign, malignant and unspecified (incl cysts and polyps) Metabolism and nutrition disorders

Metabolism and nutrition disorders Investigations

Nervous system disorders

# PT name

Dizziness

Renal impairment* Taste disorder*

Blood creatinine increased Cardiac failure

Disease progression* Constipation

Intentional product use issue Ataxia

Weight increased Cognitive disorder Oedema*

Balance disorder Oedema peripheral* Syncope

Dysgeusia Pleural effusion

Muscular weakness Renal disorder* Interstitial lung disease*

Hepatic function abnormal Dysarthria*

Cardiac failure congestive Urinary retention* Ageusia*

Ejection fraction decreased* Respiratory failure

Amnesia Pneumonitis*

Urinary incontinence*

Blood creatine phosphokinase increased* Hypoaesthesia oral*

Cardiomyopathy*

Metastases to central nervous system Troponin increased*

Neutrophil count decreased* Pain of skin*

Aspartate aminotransferase increased Arrhythmia*

Cerebral haemorrhage* Pericardial effusion* Metastases to liver Myocarditis Cardiotoxicity* Incontinence*

Left ventricular dysfunction Feeling drunk*

Skin disorder* Hypoxia Hyperkalaemia* Movement disorder* Ascites*

Pneumonia aspiration* Mood altered

Diplopia

Cerebral infarction* Orthostatic hypotension

Blood alkaline phosphatase increased Hospice care

Ischaemic stroke* Hypervolaemia* Electrolyte imbalance* Metastasis Hypernatraemia* Hyperuricaemia* Troponin I increased*

Dyslalia*

# Case number

58

35

26

25

24

21

19

19

19

18

16

15

12

11

10

10

10

9

9

8

8

8

7

7

7

7

6

6

6

6

6

6

6

5

5

4

4

4

4

4

4

4

4

4

4

4

4

3

3

3

3

3

3

3

3

3

3

3

3

3

3

3

3

3

3

3

3

# ROR (95%CI)

5.41(4.16-7.03)

14.81(10.59-20.71)

26.95(18.28-39.73)

16.79(11.31-24.94)

12.62(8.43-18.88)

7.21(4.69-11.09)

3.55(2.26-5.58)

4.44(2.83-6.98)

76.63(48.68-120.64)

3.23(2.03-5.14)

13.67(8.35-22.37)

13.92(8.37-23.15)

5.99(3.40-10.58)

5.37(2.97-9.72)

4.56(2.45-8.50)

6.83(3.67-12.72)

7.72(4.15-14.38)

3.77(1.96-7.25)

7.43(3.86-14.31)

6.44(3.22-12.91)

9.00(4.49-18.04)

10.68(5.33-21.39)

7.17(3.41-15.07)

9.39(4.47-19.74)

11.22(5.34-23.59)

15.55(7.40-32.68)

3.87(1.74-8.63)

4.61(2.07-10.28)

8.20(3.68-18.28)

9.64(4.32-21.51)

12.27(5.50-27.35)

19.30(8.65-43.06)

21.34(9.57-47.61)

15.57(6.47-37.48)

26.96(11.19-64.92)

3.44(1.29-9.18)

3.70(1.39-9.86)

3.85(1.44-10.28)

3.89(1.46-10.38)

5.46(2.04-14.56)

7.47(2.80-19.93)

8.74(3.28-23.33)

12.22(4.58-32.63)

14.98(5.61-40.00)

16.77(6.28-44.76)

24.36(9.12-65.06)

31.35(11.74-83.75)

3.26(1.05-10.11)

3.39(1.09-10.52)

4.00(1.29-12.43)

4.15(1.34-12.88)

4.57(1.47-14.19)

5.17(1.66-16.04)

5.67(1.83-17.60)

5.79(1.86-17.98)

6.40(2.06-19.87)

6.65(2.14-20.64)

7.58(2.44-23.55)

7.97(2.57-24.74)

8.15(2.63-25.31)

8.88(2.86-27.58)

10.33(3.33-32.07)

17.44(5.61-54.19)

26.74(8.60-83.12)

30.15(9.70-93.72)

68.49(21.99-213.30)

230.29(73.36-722.94)

**PRR**

5.25

14.51

26.53

16.55

12.44

7.13

3.52

4.4

75.74

3.21

13.54

13.8

5.96

5.34

4.54

6.79

7.68

3.75

7.39

6.42

8.96

10.63

7.14

9.36

11.18

15.48

3.86

4.6

8.17

9.61

12.22

19.24

21.27

15.52

26.87

3.44

3.69

3.85

3.88

5.44

7.45

8.72

12.2

14.95

16.73

24.3

31.28

3.25

3.38

4

4.14

4.56

5.16

5.66

5.78

6.39

6.64

7.57

7.96

8.14

8.87

10.31

17.41

26.69

30.1

68.36

229.86

# IC025

0.72

2.19

3.06

2.38

1.97

1.17

0.15

0.47

4.57

0.01

2.09

2.12

0.91

0.75

0.52

1.1

1.27

0.24

1.22

1.01

1.5

1.74

1.17

1.56

1.81

2.28

0.28

0.53

1.36

1.6

1.94

2.6

2.74

2.29

3.08

0.11

0.22

0.28

0.29

0.78

1.23

1.46

1.94

2.23

2.39

2.93

3.29

0.03

0.09

0.33

0.38

0.52

0.7

0.83

0.86

1.01

1.06

1.25

1.32

1.36

1.48

1.7

2.45

3.07

3.24

4.41

6.13

# SOC name

Nervous system disorders Nervous system disorders Nervous system disorders

General disorders and administration site conditions Musculoskeletal and connective tissue disorders Nervous system disorders

Neoplasms benign, malignant and unspecified (incl cysts and polyps) Investigations

Neoplasms benign, malignant and unspecified (incl cysts and polyps) Renal and urinary disorders

Surgical and medical procedures Investigations

Hepatobiliary disorders

General disorders and administration site conditions

Neoplasms benign, malignant and unspecified (incl cysts and polyps) General disorders and administration site conditions

Investigations Investigations

Neoplasms benign, malignant and unspecified (incl cysts and polyps) Neoplasms benign, malignant and unspecified (incl cysts and polyps) Respiratory, thoracic and mediastinal disorders

Neoplasms benign, malignant and unspecified (incl cysts and polyps) Neoplasms benign, malignant and unspecified (incl cysts and polyps) Neoplasms benign, malignant and unspecified (incl cysts and polyps) General disorders and administration site conditions

Surgical and medical procedures

Neoplasms benign, malignant and unspecified (incl cysts and polyps) Nervous system disorders

Investigations

Neoplasms benign, malignant and unspecified (incl cysts and polyps) Neoplasms benign, malignant and unspecified (incl cysts and polyps) General disorders and administration site conditions

General disorders and administration site conditions Nervous system disorders

Nervous system disorders Gastrointestinal disorders

Neoplasms benign, malignant and unspecified (incl cysts and polyps) Neoplasms benign, malignant and unspecified (incl cysts and polyps) General disorders and administration site conditions

Injury, poisoning and procedural complications Blood and lymphatic system disorders Infections and infestations

Hepatobiliary disorders Investigations Hepatobiliary disorders Hepatobiliary disorders Investigations

Skin and subcutaneous tissue disorders Hepatobiliary disorders

Neoplasms benign, malignant and unspecified (incl cysts and polyps) Neoplasms benign, malignant and unspecified (incl cysts and polyps) Neoplasms benign, malignant and unspecified (incl cysts and polyps) Metabolism and nutrition disorders

Neoplasms benign, malignant and unspecified (incl cysts and polyps) Nervous system disorders

Neoplasms benign, malignant and unspecified (incl cysts and polyps) Neoplasms benign, malignant and unspecified (incl cysts and polyps)

# PT name

Dizziness

Neuropathy peripheral* Paraesthesia*

Disease progression* Myalgia Hypoaesthesia*

Malignant neoplasm progression Hepatic enzyme increased Glioblastoma

Renal impairment* Therapy cessation

Alanine aminotransferase increased Hepatic function abnormal

Drug resistance* Neoplasm malignant Withdrawal syndrome*

Aspartate aminotransferase increased Transaminases increased

Metastases to lung Pancreatic carcinoma Lung disorder* Metastases to liver

Metastases to central nervous system Sarcoma

Influenza like illness* Surgery

Neoplasm progression Taste disorder*

Liver function test increased Glioma

Glioblastoma multiforme Gait inability*

Multiple organ dysfunction syndrome* Hypersomnia*

Neuralgia* Ascites* Thyroid cancer Astrocytoma Oedema*

Multiple fractures Myelosuppression* Respiratory tract infection Hepatotoxicity

Blood bilirubin increased* Hepatocellular injury* Cholestasis*

Blood alkaline phosphatase increased Sensitive skin*

Hepatic cytolysis* Metastases to lymph nodes* Lung cancer metastatic Metastases to meninges

Cell death*

Pancreatic carcinoma metastatic* Hemianaesthesia*

Soft tissue sarcoma Adenocarcinoma of salivary gland

# Case number

35

26

17

16

15

12

11

11

10

9

8

8

8

8

7

7

7

7

7

6

6

6

6

6

5

5

5

5

5

5

5

4

4

4

4

4

4

4

3

3

3

3

3

3

3

3

3

3

3

3

3

3

3

3

3

3

3

# ROR (95%CI)

3.79(2.71-5.30)

12.12(8.22-17.87)

5.68(3.52-9.16)

6.76(4.13-11.06)

5.00(3.01-8.33)

4.19(2.38-7.40)

4.48(2.48-8.11)

7.32(4.05-13.26)

310.94(165.92-582.73)

4.48(2.33-8.63)

5.12(2.56-10.26)

7.55(3.77-15.12)

10.72(5.35-21.49)

11.61(5.79-23.27)

3.78(1.80-7.95)

7.56(3.60-15.90)

8.09(3.85-17.00)

14.24(6.77-29.94)

28.18(13.40-59.26)

5.05(2.27-11.27)

5.75(2.58-12.82)

15.59(6.99-34.77)

22.25(9.97-49.63)

261.94(116.70-587.94)

3.75(1.56-9.03)

4.23(1.76-10.17)

4.48(1.86-10.79)

6.79(2.82-16.35)

8.00(3.32-19.25)

372.03(153.03-904.44)

416.83(171.25-1014.59)

3.60(1.35-9.62)

5.00(1.87-13.34)

6.96(2.61-18.58)

7.02(2.63-18.74)

7.07(2.65-18.86)

7.93(2.97-21.17)

611.97(225.11-1663.68)

3.23(1.04-10.01)

4.03(1.30-12.50)

4.21(1.36-13.08)

5.27(1.70-16.38)

5.48(1.77-17.03)

7.00(2.25-21.73)

7.20(2.32-22.35)

7.39(2.38-22.94)

8.44(2.72-26.21)

10.38(3.34-32.25)

11.69(3.76-36.31)

20.81(6.70-64.65)

60.91(19.58-189.52)

62.51(20.09-194.50)

62.84(20.20-195.53)

67.75(21.77-210.84)

110.92(35.59-345.73)

311.75(99.32-978.58)

2805.77(821.28-9585.45)

**PRR**

3.71

11.91

5.62

6.69

4.96

4.16

4.45

7.27

308.64

4.46

5.1

7.51

10.66

11.55

3.77

7.53

8.05

14.17

28.04

5.04

5.73

15.52

22.15

260.78

3.74

4.21

4.47

6.77

7.97

370.65

415.28

3.6

4.99

6.95

7

7.05

7.91

610.15

3.22

4.02

4.2

5.26

5.47

6.99

7.18

7.37

8.42

10.36

11.67

20.76

60.78

62.37

62.7

67.6

110.68

311.06

2799.51

# IC025

0.22

1.9

0.82

1.07

0.64

0.39

0.49

1.19

6.57

0.49

0.68

1.24

1.75

1.86

0.25

1.24

1.34

2.16

3.14

0.66

0.85

2.29

2.8

6.33

0.23

0.41

0.49

1.09

1.33

6.82

6.98

0.18

0.65

1.13

1.14

1.15

1.32

7.51

0.02

0.34

0.4

0.73

0.78

1.14

1.18

1.21

1.41

1.7

1.88

2.71

4.25

4.29

4.29

4.4

5.11

6.57

9.43
