# Supplementary material for: From genomic spectrum of NTRK genes to adverse effects of its inhibitors, a comprehensive genome-based and real-world pharmacovigilance analysis
Source: Front Pharmacol. 2024 Jan 31;15:1329409. doi: 10.3389/fphar.2024.1329409 (PMC10864613; doi:10.3389/fphar.2024.1329409)
Supplement: Supplementary file 8 [file Table6.docx]

| **Entrectinib** | | | | | | | | | | |
| --- | --- | --- | --- | --- | --- | --- | --- | --- | --- | --- |
| SOC name | case number | min | max | median | IQR | q1 | q3 | mean | sd | se |
| Nervous system disorders | 167 | 0 | 740 | 7 | 11 | 2 | 13 | 22.07 | 70.34 | 5.44 |
| General disorders and administration site conditions | 103 | 0 | 1425 | 10 | 26.5 | 2 | 28.5 | 69.59 | 198.74 | 19.58 |
| Investigations | 84 | 0 | 328 | 9.5 | 24.25 | 3 | 27.25 | 28.13 | 56.26 | 6.14 |
| Gastrointestinal disorders | 59 | 0 | 635 | 4 | 19.5 | 1 | 20.5 | 40.36 | 117.63 | 15.31 |
| Renal and urinary disorders | 54 | 0 | 263 | 9.5 | 32.25 | 3 | 35.25 | 34.65 | 59.86 | 8.15 |
| Respiratory, thoracic and mediastinal disorders | 53 | 0 | 694 | 10 | 28 | 2 | 30 | 72.62 | 142.6 | 19.59 |
| Cardiac disorders | 50 | 0 | 932 | 10 | 34.5 | 3 | 37.5 | 59.74 | 165.18 | 23.36 |
| Injury, poisoning and procedural complications | 44 | 0 | 403 | 13.5 | 97.25 | 1 | 98.25 | 73.59 | 112.09 | 16.9 |
| Infections and infestations | 36 | 0 | 1765 | 12.5 | 278.5 | 7 | 285.5 | 233.19 | 410.8 | 68.47 |
| Musculoskeletal and connective tissue disorders | 31 | 0 | 1300 | 6 | 122.5 | 1.5 | 124 | 152.35 | 307.08 | 55.15 |
| Metabolism and nutrition disorders | 30 | 0 | 246 | 9.5 | 18.75 | 2.25 | 21 | 35 | 63.33 | 11.56 |
| Psychiatric disorders | 24 | 0 | 92 | 1 | 9.25 | 0 | 9.25 | 11.25 | 25.49 | 5.2 |
| Skin and subcutaneous tissue disorders | 21 | 0 | 740 | 10 | 11 | 2 | 13 | 84.05 | 219.16 | 47.82 |
| Vascular disorders | 14 | 1 | 195 | 12.5 | 108.75 | 3.25 | 112 | 60.71 | 80.52 | 21.52 |
| Neoplasms benign, malignant and unspecified (incl cysts and polyps） | 13 | 0 | 740 | 17 | 156 | 8 | 164 | 126.77 | 214.35 | 59.45 |
| Eye disorders | 9 | 0 | 363 | 4 | 16 | 1 | 17 | 55.78 | 120.3 | 40.1 |
| Hepatobiliary disorders | 9 | 0 | 677 | 12 | 19 | 8 | 27 | 87.67 | 221.32 | 73.77 |
| Blood and lymphatic system disorders | 7 | 0 | 131 | 23 | 34.5 | 11.5 | 46 | 38.43 | 44.82 | 16.94 |
| Endocrine disorders | 4 | 1 | 386 | 4.5 | 101.5 | 1 | 102.5 | 99 | 191.36 | 95.68 |
| Surgical and medical procedures | 4 | 4 | 99 | 10 | 25.25 | 7.75 | 33 | 30.75 | 45.6 | 22.8 |
| **Larotrectinib** | | | | | | | | | | |
| SOC name | case number | min | max | median | IQR | q1 | q3 | mean | sd | se |
| Nervous system disorders | 56 | 0 | 588 | 9 | 70.25 | 3.75 | 74 | 85.29 | 152.21 | 20.34 |
| General disorders and administration site conditions | 49 | 0 | 588 | 20 | 109 | 5 | 114 | 100.12 | 154.24 | 22.03 |
| Neoplasms benign, malignant and unspecified (incl cysts and polyps） | 47 | 0 | 545 | 101 | 161 | 16 | 177 | 120.43 | 120.35 | 17.55 |
| Investigations | 32 | 0 | 545 | 24 | 47.75 | 6.75 | 54.5 | 62.5 | 109.79 | 19.41 |
| Gastrointestinal disorders | 31 | 0 | 399 | 21 | 45 | 8 | 53 | 80.03 | 129.31 | 23.22 |
| Injury, poisoning and procedural complications | 30 | 0 | 980 | 36 | 165.25 | 4 | 169.25 | 106.7 | 189.45 | 34.59 |
| Musculoskeletal and connective tissue disorders | 21 | 0 | 436 | 53 | 197 | 3 | 200 | 127.33 | 150.23 | 32.78 |
| Respiratory, thoracic and mediastinal disorders | 21 | 0 | 436 | 10 | 72 | 3 | 75 | 68.81 | 116.11 | 25.34 |
| Hepatobiliary disorders | 19 | 4 | 193 | 28 | 19 | 14.5 | 33.5 | 35.42 | 40.88 | 9.38 |
| Skin and subcutaneous tissue disorders | 13 | 0 | 207 | 8 | 9 | 1 | 10 | 32.85 | 64.09 | 17.77 |
| Metabolism and nutrition disorders | 11 | 14 | 314 | 28 | 82.5 | 21 | 103.5 | 79.64 | 95.92 | 28.92 |
| Psychiatric disorders | 11 | 0 | 119 | 4 | 24 | 4 | 28 | 26.45 | 40.37 | 12.17 |
| Blood and lymphatic system disorders | 9 | 0 | 193 | 90 | 133 | 14 | 147 | 80.44 | 70.52 | 23.51 |
| Renal and urinary disorders | 9 | 0 | 242 | 21 | 14 | 14 | 28 | 41.89 | 75.58 | 25.19 |
| Infections and infestations | 8 | 28 | 494 | 242 | 334.25 | 64.75 | 399 | 238.63 | 181.87 | 64.3 |
| Surgical and medical procedures | 5 | 0 | 980 | 54 | 492 | 2 | 494 | 306 | 429.88 | 192.25 |
| Vascular disorders | 3 | 0 | 28 | 28 | 14 | 14 | 28 | 18.67 | 16.17 | 9.33 |

| **Entrectinib** | | |
| --- | --- | --- |
| day group | case number | percentage |
| <=30 | 147 | 0.6049 |
| 31-60 | 26 | 0.107 |
| 61-90 | 10 | 0.0411 |
| 91-180 | 17 | 0.07 |
| 181-360 | 18 | 0.0741 |
| >360 | 25 | 0.1029 |
| **Larotrectinib** | | |
| day group | case number | percentage |
| <=30 | 53 | 0.469 |
| 31-60 | 13 | 0.115 |
| 61-90 | 9 | 0.0797 |
| 91-180 | 16 | 0.1416 |
| 181-360 | 12 | 0.1062 |
| >360 | 10 | 0.0885 |
